# Profiling the tumor-resident microbiota in small cell lung cancer and its influence on clinical outcomes

**DOI:** 10.1186/s12967-026-08109-x

**Published:** 2026-04-14

**Authors:** Wengang Zhang, Wencheng Zhao, Li Ye, Hao Wang, Zhimin Chen, Xinyue Liu, Yujie Li, Qianqian Zhang, Huixian Zhang, Yujin Liu, Xuyang Chen, Shiyin Chen, Jialin Zeng, Runze Huang, Yuhang Li, Yayi He

**Affiliations:** Department of Medical Oncology, Shanghai Pulmonary Hospital, School of Medicine, Tongji University, Shanghai, 200433 China

**Keywords:** Small cell lung cancer, Tumor microbiota, Clostridium, *Lactobacillus*

## Abstract

**Background:**

Increasing evidence has confirmed the existence of resident microbial communities within solid tumors and indicates that the tumor microbiota may represent a novel component of the tumor microenvironment, actively regulating cancer initiation, progression, metastasis, and therapeutic responsiveness. However, the role of tumor microbiota in small cell lung cancer (SCLC) has not been well explored.

**Methods:**

Tumor samples were collected from 71 patients with SCLC at Shanghai Pulmonary Hospital between 2019 and 2023. A comprehensive analysis of the tumor-resident microbiota in SCLC was conducted using 16 S rRNA sequencing. The bacterial communities were profiled, and their correlation with clinical outcomes was assessed.

**Results:**

A total of 28 phyla, 79 classes, 135 orders, 251 families, and 428 genera were identified, revealing a diverse tumor microbiota in SCLC. It was shown that tumor microbiota varied markedly among patients. SCLC patients with a smoking history exhibited distinct tumor microbiota, with significantly higher abundances of *Brevundimonas* and *Gemmatimonadetes*. When patients were stratified by progression-free survival (PFS) into long-PFS (L-PFS) and short-PFS (S-PFS) cohorts, their tumor microbiota segregated distinctly. LEfSe analysis showed that *Lactobacillus*, *Clostridium*, *Rothia*, and *Staphylococcus* were selectively enriched in the L-PFS group, whereas *Stenotrophomonas*, *Cetobacterium*, and *Aerococcus* dominated the S-PFS group. Kaplan–Meier analysis confirmed that carriage of *Lactobacillus*, *Clostridium*, or *Staphylococcus* was associated with prolonged survival relative to negative status, while positivity for *Stenotrophomonas*, *Cetobacterium*, or *Aerococcus* conferred a reduction in survival. Subsequent response-stratified analysis revealed that *Clostridium*- and *Lactobacillus*-positive SCLC was associated with a significantly higher response rate. Conversely, positivity for *Methylobacterium*, *Pelomonas*, *Ralstonia*, *Bradyrhizobium*, *Variovorax*, *Microbacterium*, *Comamonas*, or *Sphingomonas* markedly reduced response. The intersection of survival and response results identify *Clostridium* and *Lactobacillus* as promising prognostic tumor microbiota markers in SCLC. In vitro assays demonstrated that the *Clostridium* and *Lactobacillus* metabolites, butyrate and lactic acid, lacked direct cytotoxicity against SCLC cells. However, in the syngeneic mouse model, systemic supplementation of butyrate or lactic acid significantly potentiated the anti-tumor efficacy of standard chemotherapy. Notably, flow cytometric analysis revealed that this in vivo synergistic effect was closely associated with a profound increase in CD8 + T cell infiltration within the tumor microenvironment. Furthermore, integrating these two tumor microbiota with key clinical variables (sex, age, smoking, stage, radiotherapy), we constructed three models—therapeutic-response, 1-year PFS, and 1-year overall survival—that maintained robust performance in both training and validation cohorts.

**Conclusions:**

In conclusion, the tumor-resident microbiota constitutes a critical component of the SCLC tumor microenvironment, exerting profound influence on the therapeutic response and patient prognosis. In detail, *Clostridium* and *Lactobacillus*—two pivotal tumor-resident taxa significantly linked to enhanced therapeutic responses and favorable prognosis—indicate their potential as predictive biomarkers for treatment outcomes and patient prognosis, and highlight them as candidate targets for microbiome-directed therapeutic strategies against SCLC, which warrants further functional validation.

**Graphical Abstract:**

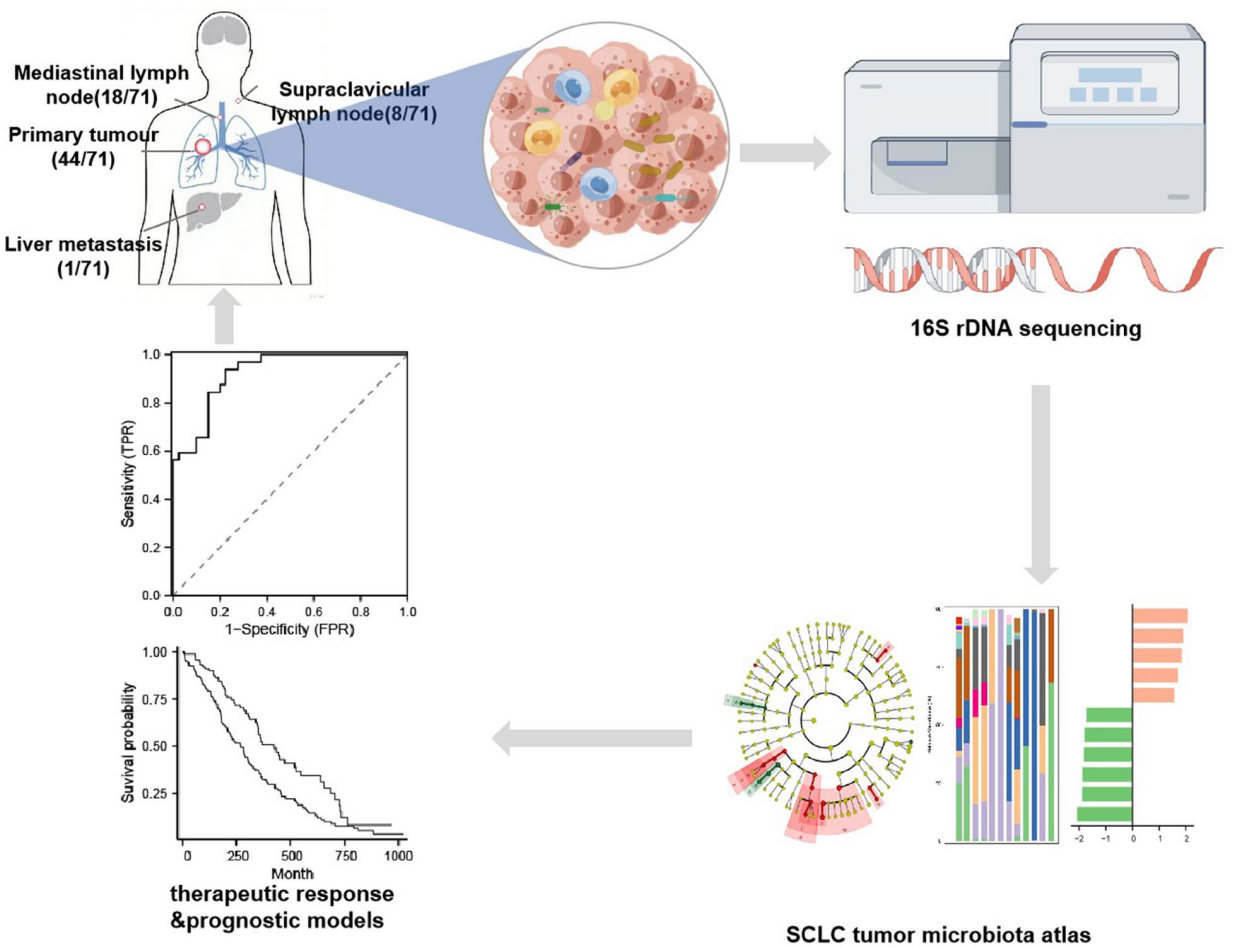

**Supplementary Information:**

The online version contains supplementary material available at 10.1186/s12967-026-08109-x.

## Highlights

First comprehensive tumor microbiota profiling in SCLC patients.

Tumor microbiota correlates with survival and therapy response in SCLC, with *Clostridium*- and *Lactobacillus*-positive patients experiencing better outcomes.

Tumor microbiota-clinical variable models effectively predict treatment response and survival.

**Supplementary Information:**

The online version contains supplementary material available at 10.1186/s12967-026-08109-x.

## Introduction

Small cell lung cancer (SCLC) is the most aggressive and lethal form of lung cancer, accounting for approximately 15% of all lung cancer cases [1]. Characterized by rapid tumor growth, early metastasis, and a high relapse propensity, SCLC is rarely treated with surgery according to NCCN guidelines, except in the T1-2N0M0 stage [2]. Statistics show that nearly two-thirds of SCLC have distant metastasis at initial diagnosis [1]. For limited- or extensive-stage SCLC beyond T1-2N0M0, the standard first-line treatment has been chemotherapy with or without radiotherapy, to which most patients initially respond well [1]. Despite this, the majority of patients develop resistance within approximately 6 months, with a median survival time of less than 1 year [3].

Over the past several decades, extensive research has sought to elucidate the molecular mechanisms underlying drug resistance in SCLC [4, 5]. Current evidence supports a multifactorial model that involves enhanced DNA damage repair, upregulation of drug-efflux and detoxification systems, evasion of apoptotic pathways, epigenetic remodeling, tumor heterogeneity and clonal evolution, and resistance acquired through tumor microenvironment (TME) interactions [6–8]. Of particular note is the TME, which provides a nurturing soil for tumor cell survival and has been increasingly recognized in recent years as playing a critical role in tumor initiation and progression [9]. Contemporary research on therapy resistance mediated by the TME has largely centred on immune cell diversity and infiltration, cancer-associated fibroblasts and extracellular matrix remodelling, dysregulated angiogenesis and hypoxia, as well as soluble signalling molecules and metabolic cues [10].

Mounting evidence has unequivocally established the presence of intratumoral microbiota across a diverse spectrum of human cancers [11]. The tumor-resident microbiota, which colonizes tumor tissues, may represent a novel, critical component of the TME—orchestrating key processes including oncogenesis, tumor progression, and therapeutic responsiveness by metabolite production, immune modulation, and direct interactions with tumor cells [12]. In a pan-cancer metastasis cohort, Emile et al. detected tumor-resident bacterial DNA and demonstrated that bacterial diversity correlates with features of cellular and molecular tumor immunity [13]. Specifically, a higher abundance of *Fusobacterium* was associated with poor response to immunotherapy in non-small cell lung cancer (NSCLC) [13]. Susan et al. integrated spatial transcriptomics, spatial proteomics, and single-cell sequencing to systematically elucidate the spatial distribution of intratumoral microbiota and its influence on tumor heterogeneity, the immune microenvironment, and cancer cell behavior [14]. Besides, Chen et al. provided evidence that intracellular tumor-resident microbiota promotes metastatic colonization in breast cancer [15]. Originating from the bronchial–alveolar epithelium, lung cancers are in continuous contact with the external environment and are thus predisposed to harbour a complex intratumoral microbiota that may orchestrate tumor proliferation, metastasis, and therapeutic responsiveness [11]. Nevertheless, the characterization of the tumor microbiome in SCLC and its influence on therapeutic efficacy and patient survival remains inadequate and merits further investigation.

In this study, we aimed to comprehensively characterize the tumor-resident microbiota in SCLC via 16 S rRNA sequencing and to evaluate its potential impact on chemotherapy response. Furthermore, we sought to identify microbial features associated with patient prognosis and to construct a prognostic prediction model. These results will provide important insights into the mechanisms of chemoresistance and facilitate the development of microbiota-targeted therapeutic strategies for SCLC.

## Methods

### Participant recruitment and specimen acquisition

Seventy-one SCLC tumor samples (70 obtained prior to any systemic therapy and one collected at the time of acquired resistance) were consecutively procured from patients treated at Shanghai Pulmonary Hospital, Tongji University, between January 2019 and December 2023. All patients received first-line chemotherapy with or without concurrent immunotherapy. The inclusion criteria were as follows: (1) histologically or cytologically confirmed SCLC; (2) stage IIIA–IVB disease according to the 8th edition of the American Joint Committee on Cancer staging system; (3) at least one measurable lesion as defined by Response Evaluation Criteria in Solid Tumors (RECIST) version 1.1; and (4) an Eastern Cooperative Oncology Group (ECOG) performance status of 0 or 1.

### Efficacy evaluation

Tumor objective response was evaluated in accordance with the RECIST guidelines. The response criteria for target lesions were defined as follows: complete response (CR), characterized by the disappearance of all target lesions with the short-axis diameter of target lymph nodes reduced to < 10 mm; partial response (PR), referring to a ≥ 30% reduction in the sum of the longest diameters of target lesions compared with baseline; progressive disease (PD), defined as a ≥ 20% increase in the sum of the longest diameters of target lesions (with an absolute increase of ≥ 5 mm) using the smallest sum of diameters recorded during the study as the reference, or the emergence of new malignant lesions; and stable disease (SD), indicating a response that did not meet the criteria for PR or PD.

### In vitro cell viability assay

To evaluate the direct tumor-cell-intrinsic effects of microbiota-derived metabolites, the murine SCLC cell line RPS1 (generously provided by Prof. Hongbin Ji, Westlake University, China) was seeded into 96-well plates. Cells were exposed to butyrate (1 mM) or lactic acid (5 mM), alone or combined with cisplatin (2 μm). Following a 24-h incubation, cell viability was quantified via CCK-8 assay per the manufacturer’s protocol, utilizing PBS as the vehicle control. All assays were performed in biological triplicates.

### In vivo syngeneic tumor models and treatments

To evaluate the in vivo synergistic effects of microbiota-derived metabolites and chemotherapy, a syngeneic SCLC mouse model was established. Briefly, 1 × 10^6^ RPS1 cells were subcutaneously injected into the right flank of 6- to 8-week-old male C57BL/6 mice. When the tumor volume reached 100–200 mm³, the mice were randomly assigned into four treatment groups (*n* = 5 per group): (1) Control (Vehicle); (2) E/P regimen alone; (3) E/P + Lactic acid; and (4) E/P + Butyrate. For the standard chemotherapy regimen (E/P), mice were treated intraperitoneally with cisplatin (6 mg/kg) on day 1, and etoposide (10 mg/kg) on days 1 to 3 of each weekly cycle [16]. For metabolite supplementation, mice received intraperitoneal injections of sodium butyrate (200 mg/kg) [17]or lactic acid (250 mg/kg) every other day throughout the experiment. The control group received equivalent volumes of sterile PBS [18]. Tumor dimensions and mouse body weights were monitored every other day. Tumor volume was calculated using the formula: *V = 0.5×length×width*^*2*^. At the experimental endpoint, the mice were euthanized, and the tumors were excised, photographed, weighed, and further processed for flow cytometric analysis of the immune microenvironment.

### Genomic DNA extraction, microbial library construction, and sequencing

Total microbial DNA was extracted, and its concentration and quality were assessed using a NanoDrop spectrophotometer and agarose gel electrophoresis. The V3–V4 hypervariable region of the 16 S rRNA gene was amplified with barcoded primers and Pfu high-fidelity DNA polymerase (TransGen Biotech) under a minimal cycle number to reduce amplification bias. Negative controls were included to monitor contamination. Amplicons were purified with VAHTS DNA Clean Beads (Vazyme) and quantified with the Quant-iT PicoGreen dsDNA Assay Kit on a BioTek FLx800 microplate reader. Based on the quantification results, amplicons were pooled at equimolar ratios. Sequencing libraries were prepared using the Illumina TruSeq Nano DNA LT Library Prep Kit, including end-repair, A-tailing, adapter ligation, and an 8-cycle enrichment PCR. Library purification and size selection were performed with AMPure XP beads and 2% agarose gel electrophoresis. Final libraries were evaluated on an Agilent Bioanalyzer with the High Sensitivity DNA Kit and quantified with the Quant-iT PicoGreen system. Libraries meeting quality criteria were denatured and sequenced on either an Illumina MiSeq (MiSeq Reagent Kit v3, 600 cycles) or NovaSeq 6000 (NovaSeq 6000 SP Reagent Kit, 500 cycles) platform with an optimal insert size of 200–450 bp to ensure high read quality.

### Microbiota bioinformatics analysis

Paired-end raw sequencing data were first subjected to quality control. Primer sequences were trimmed using cutadapt. Reads were then processed in QIIME2. Denoising, merging of paired-end reads, and amplicon sequence variant (ASV) inference were performed with DADA2; chimeric sequences were subsequently removed. Taxonomy assignment of ASVs was carried out with the SILVA reference database. A phylogenetic tree was constructed using MAFFT and FastTree for phylogenetic-based diversity analyses. Alpha diversity and beta diversity were calculated after rarefying all samples to an even sequencing depth. Differences in alpha diversity between groups were assessed using the Kruskal–Wallis test or Wilcoxon rank-sum test, where appropriate. β-diversity patterns were rendered by four complementary visualization strategies: (i) principal component analysis (PCA) of centred log-ratio-transformed genus abundances; (ii) principal coordinates analysis (PCoA) based on Bray–Curtis and both weighted and unweighted UniFrac distances; (iii) non-metric multidimensional scaling (NMDS) on the same distance matrices; and (iv) hierarchical clustering using the unweighted pair-group method with arithmetic mean (UPGMA) applied to Bray–Curtis dissimilarity. Differentially abundant taxa were identified using the Linear discriminant analysis Effect Size (LEfSe) method (Kruskal–Wallis test *p* < 0.05, logarithmic LDA score threshold > 2.0). The results were further profiled with STAMP. Microbial status was defined as “positive” if the specific taxon was detected (read count > 0), and “negative” if it was completely absent.

### Construction of prediction model

Seventy-one patients were randomly allocated to training and validation sets in a 7:3 ratio. Regarding treatment response, the prediction model integrating tumor microbiota (Clostridium and Lactobacillus) and clinical variables (sex, age, smoking status, tumor stage, and radiotherapy) was constructed using multivariable logistic regression in the training set and validated in the validation set. Regarding survival, 12-month PFS and 12-month OS prediction models were built with the same variables using multivariable Cox regression and subsequently validated in the validation set.

### Statistical analysis

Statistical analyses and graphical representations were conducted using GraphPad Prism 9.0 (GraphPad Software Inc., La Jolla, CA, USA) and R (version 4.1.3). Continuous variables were compared with unpaired t-tests (normal data) or Wilcoxon rank-sum tests (non-normal data). Categorical variables were analyzed with Pearson’s χ² test when all expected counts were ≥ 5 and total *n* ≥ 40; otherwise, Fisher’s exact test was used. Survival analyses were conducted using the Kaplan-Meier method and compared using the log-rank test. Two-sided *P* < 0.05 indicated significance.

## Results

### Overview of the clinicopathological characteristics and clinical outcomes of enrolled SCLC patients

**Fig. 1:**
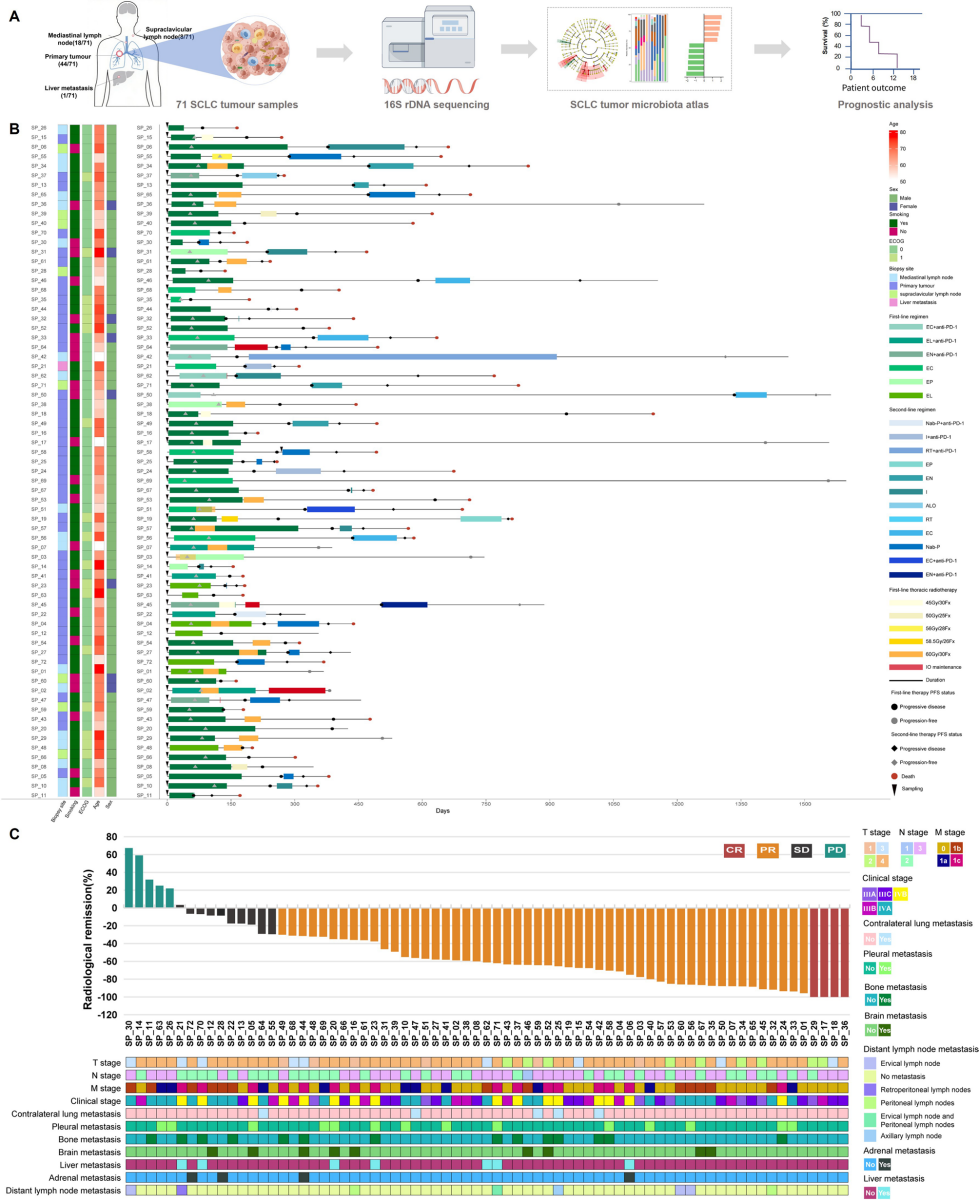
Demographic and clinical characteristics of the 71 enrolled SCLC patients.** A**. Flow chart for this study. **B**. Waterfall plots of radiological regression of target lesions for patients following treatment. **C**. Schematic diagram illustrating the disease course of the 71 studied SCLC patients. The left panel depicts a heatmap of individual baseline clinical characteristics (sex, age, ECOG performance status, smoking history, and biopsy site). The right panel displays a swimmer plot outlining the longitudinal treatment history for each patient, including sequences and durations of systemic therapy (first-line therapy, second-line therapy, and radiotherapy), alongside key clinical milestones such as time to progression and survival status

Figure 1A presents the study workflow. To characterize the tumor microbiota in SCLC, we prospectively recruited a real-world cohort of 71 patients with histologically confirmed SCLC who received chemotherapy alone or in combination with immunotherapy. As shown in Fig. 1B, individual patient data are detailed, encompassing clinicopathological characteristics and clinical outcomes—including sequences and durations of systemic treatments (first-line therapy, second-line therapy, and radiotherapy)—along with key clinical milestones such as time to progression and overall survival status. Among 71 enrolled patients (mean age, 65.7 ± 7.2 years; 88.7% male), 54.9% had extensive-stage and 45.1% limited-stage disease; 66.2% were ever-smokers. Biopsies were obtained from primary tumors (62.0%), mediastinal lymph nodes (25.4%), supraclavicular lymph nodes (11.3%), and liver metastases (1.4%). Treatment-naïve samples predominated (98.6%, 70/71), with one post-treatment sample (1.4%). Waterfall plots depict the radiological regression of target lesions across all patients, with best overall responses as follows: partial response in 52 (73.2%), stable disease in 10 (14.1%), complete response in 4 (5.6%), and progressive disease in 5 (7.0%) (Fig. 1C).

### Profiling the tumor microbiota landscape in SCLC

**Fig. 2:**
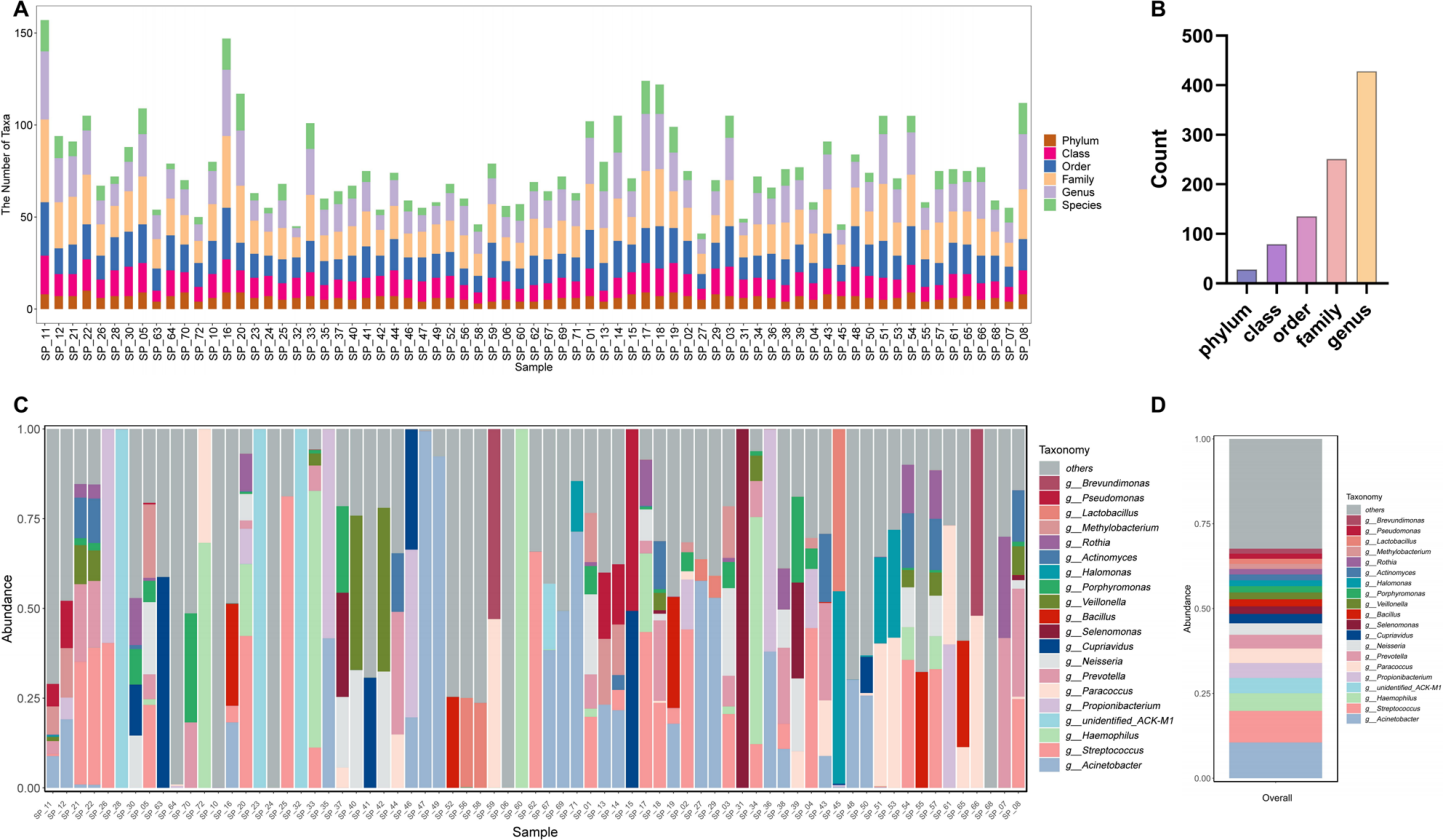
Comprehensive characterization of the tumor microbiota landscape in SCLC. **A**. Stacked bar chart showing the number of ASVs/OTUs assigned at each taxonomic rank for individual patients (*n* = 71). The x-axis displays individual samples arranged by name. The y-axis represents the count of features successfully annotated at each taxonomic level (Phylum, Class, Order, Family, Genus, and Species). The relative height of segments within each bar indicates the resolution of taxonomic classification, where greater proportions of classifications at finer taxonomic levels (e.g., Genus and Species) correspond to higher annotation resolution. **B**. Pooled bar plot presenting the aggregate count of microbial features annotated at each taxonomic level (Phylum through Species) from the combined 16 S rRNA sequencing analysis of 71 tumor samples. **C**. Tumor microbiota composition profiling of the top 20 taxa at genus level across all samples. **D**. Overall relative abundance of the top 20 bacterial genera in a pooled analysis of tumor microbiota from the enrolled 71 SCLC patients

Comprehensive profiling of the tumor microbiota in SCLC was performed via 16 S rRNA sequencing. We first quantified the number of ASVs/OTUs at each taxonomic level (phylum, class, order, family, and genus) for each individual patient (Fig. 2A) and then visualized the pooled counts across the taxonomic hierarchy (Fig. 2B). The compositional landscape of tumor microbiota is visualized in Fig. 2C through stacked bar plots, which depict the top 20 genera for each patient. Parallel analyses were also conducted to characterize the tumor microbiota at the phylum (Figure S1A), class (Figure S1B), order (Figure S1C), and family (Figure S1D) taxonomic ranks. Based on pooled analysis of the tumor microbiota from 71 SCLC samples, we calculated the relative abundance of each bacterial taxon. The top 20 most abundant genera (e.g., *Acinetobacter*, *Streptococcus*, *Haemophilus*, and *Lactobacillus*) are presented in Fig. 2D. Additionally, pooled analyses were conducted across phylum, class, order, and family levels, with the top 20 taxa displayed as stacked bar plots (Figure S2A-D).

### Comparison of tumor microbiota profiles between long progression-free survival (L-PFS) and short-PFS (S-PFS) groups

**Fig. 3:**
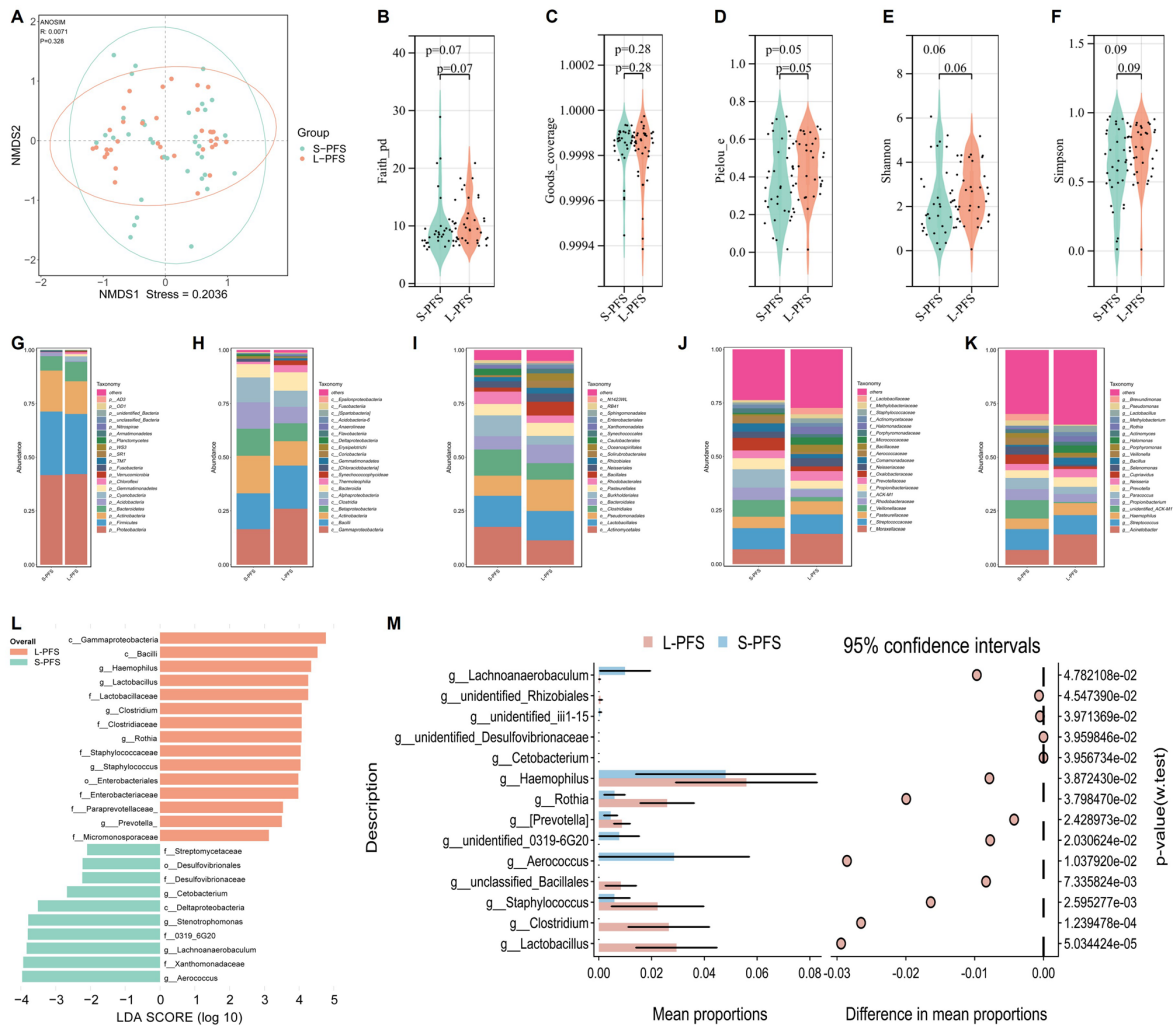
Comparison of tumor microbiota profiles between long progression-free survival (L-PFS) and short PFS (S-PFS) in SCLC. **A**. Intergroup distance display based on NMDS algorithm. **B-F**. Comparison of bacterial community α-diversity indices—including Faith_pd, Goods_coverage, Pielou_e, Shannon, and Simpson—between L-PFS and S-PFS groups. **G**. Bar plot comparing phylum-level tumor microbiota profiles between L-PFS and S-PFS groups. **H**. Bar plot comparing class-level tumor microbiota profiles between L-PFS and S-PFS groups. **I**. Bar plot comparing order-level tumor microbiota profiles between L-PFS and S-PFS groups. **J**. Bar plot comparing family-level tumor microbiota profiles between L-PFS and S-PFS groups. **K**. Bar plot comparing genus-level tumor microbiota profiles between L-PFS and S-PFS groups. **L**. Differential abundance of tumor microbiota between L-PFS and S-PFS groups, as determined by LEfSe analysis (LDA score > 2 and *P* < 0.05). **M**. Comparison of relative taxon abundance at the genus level between L-PFS and S-PFS groups, as determined by the Wilcoxon rank-sum test

Beta-diversity was evaluated using NMDS to visualize the overall composition of the tumor microbiota, with no clear separation being observed between the L-PFS and S-PFS groups (Fig. 3A). The α-diversity of the bacterial community was assessed and found to be comparable between the two groups (Fig. 3B-F). Furthermore, the proportion of different tumor microbiota in the L-PFS and S-PFS groups was calculated, respectively, at different taxonomic levels, including phylum (Fig. 3G), class (Fig. 3H), order (Fig. 3I), family (Fig. 3J), and genus (Fig. 3K). LEfSe analysis was conducted to identify potentially differential tumor microbiota between the L-PFS and S-PFS groups. As a result, it was revealed that *Lactobacillus*, *Clostridium*, *Rothia*, *Staphylococcus*, etc., were significantly enriched in the L-PFS group, while *Stenotrophomonas*, *Cetobacterium*, *Aerococcus*, etc., were predominantly enriched in the S-PFS group (Fig. 3L). Wilcoxon rank-sum tests at the genus level yielded largely consistent results, with pronounced enrichment of *Lactobacillus*, *Clostridium*, *Rothia*, and *Staphylococcus* in the L-PFS group (Fig. 3M).

### Survival analysis based on differential tumor microbiota

**Fig. 4:**
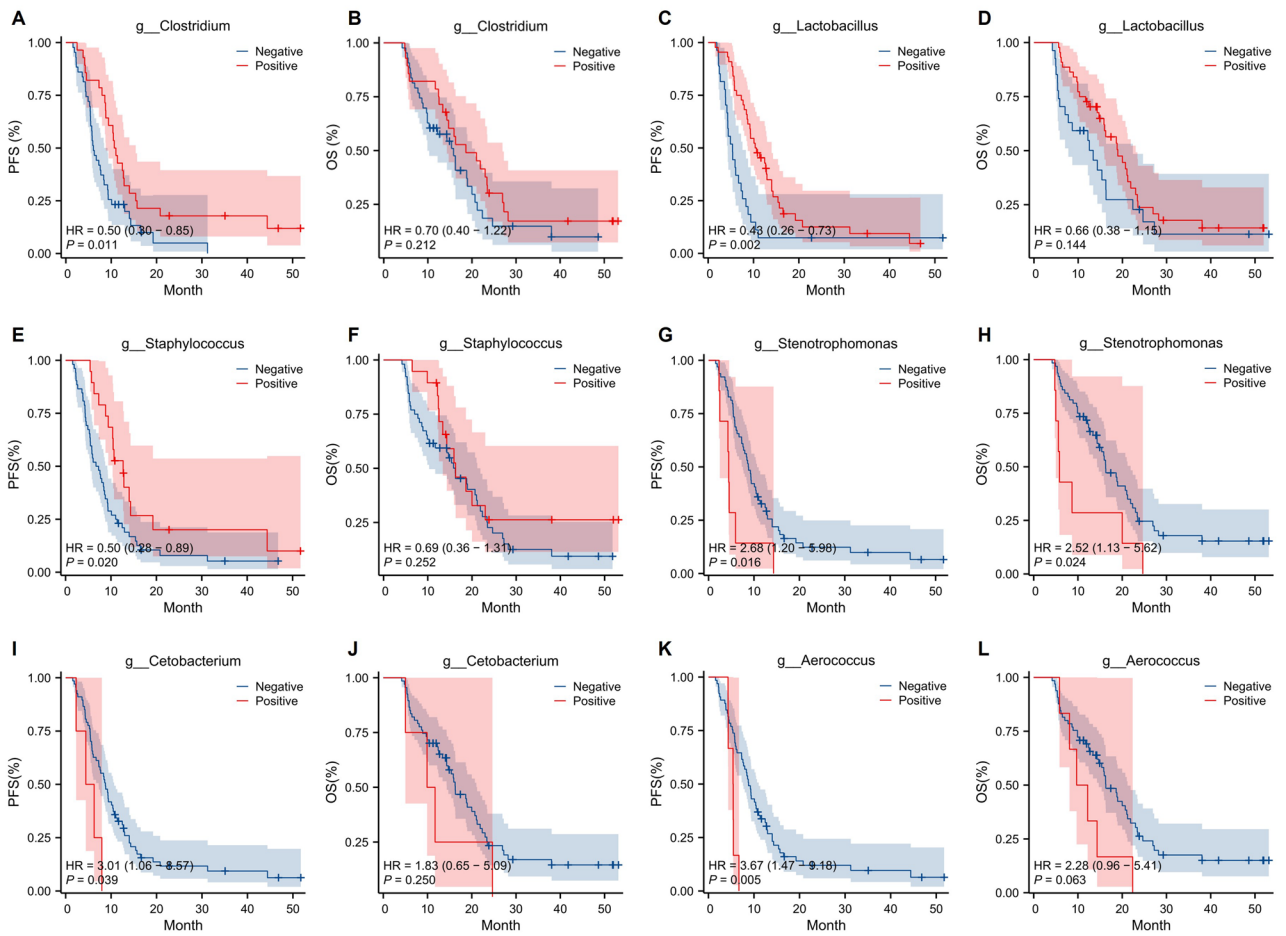
Survival analysis based on differential tumor microbiota status (negative vs. positive). **A**. Comparison of progression-free survival (PFS) according to *Clostridium* status (negative vs. positive). **B**. Comparison of overall survival (OS) according to *Clostridium* status (negative vs. positive). **C**. Comparison of PFS according to *Lactobacillus* status (negative vs. positive). **D**. Comparison of OS according to *Lactobacillus* status (negative vs. positive). **E**. Comparison of PFS according to *Staphylococcus* status (negative vs. positive). **F**. Comparison of OS according to *Staphylococcus* status (negative vs. positive). **G**. Comparison of PFS according to *Stenotrophomonas* status (negative vs. positive). **H**. Comparison of OS according to *Stenotrophomonas* status (negative vs. positive). **I**. Comparison of PFS according to *Cetobacterium* status (negative vs. positive). **J**. Comparison of OS according to *Cetobacterium* status (negative vs. positive). **K**. Comparison of PFS according to *Aerococcus* status (negative vs. positive). **L**. Comparison of OS according to *Aerococcus* status (negative vs. positive)

To further explore the prognostic impact of the identified differential tumor microbiota, survival analysis was conducted by stratifying patients according to microbial abundance (high vs. low) or status (negative vs. positive). Firstly, some tumor microbiota taxa associated with a favorable prognosis were identified. Survival analysis revealed that *Clostridium*-positive patients had significantly prolonged PFS (Fig. 4A) and a trend toward longer overall survival (OS) (Fig. 4B) compared to *Clostridium*-negative patients. Further analysis stratified by median abundance among *Clostridium*-positive patients showed that high abundance was associated with significantly better survival outcomes than low abundance (Figure S3A-B), particularly in OS (*P* = 0.03, HR = 0.39, 95% CI: 0.17–0.91, Figure S3B). Similar to *Clostridium*, survival analysis revealed that patients positive for *Lactobacillus* (Fig. 4C-D) or Staphylococcus (Fig. 4E-F) exhibited significantly prolonged survival compared to their negative counterparts. Abundance-based survival analysis is shown in Figure S3C-F. Secondly, a subset of tumor microbiota was found to be associated with an unfavorable prognosis. Specifically, patients positive for *Stenotrophomonas* (Fig. 4G-H), *Cetobacterium* (Fig. 4I-J), and *Aerococcus* (Fig. 4K-L) showed shorter survival compared to their negative counterparts. Further survival analysis was conducted by stratifying the positive patients into high- and low-abundance groups according to the median value; however, due to the limited sample size, no significant difference was observed (Figure S3G-J). Thirdly, analysis of other differential tumor microbiota, including *Prevotella* (Figure S4A-D), *Lachnoanaerobaculum* (Figure S4E-H), *Haemophilus* (Figure S4I-L), and *Rothia* (Figure S4M-P), revealed no significant association with patient prognosis. Finally, to evaluate the independent prognostic value of the tumor microbiota, we performed multivariate Cox regression analyses adjusting for stage, radiotherapy, sex, etc. (Tables S1-12). For PFS, *Clostridium* (Table S3), *Lactobacillus* (Table S4), *Staphylococcus* (Table S5), and *Stenotrophomonas* (Table S6) were identified as independent prognostic factors. However, no statistically significant association with OS was observed for any of these microbiota (Tables S7-12), potentially due to the limited sample size.

### Comparison of tumor microbiota profiles between treatment responders and non-responders

**Fig. 5:**
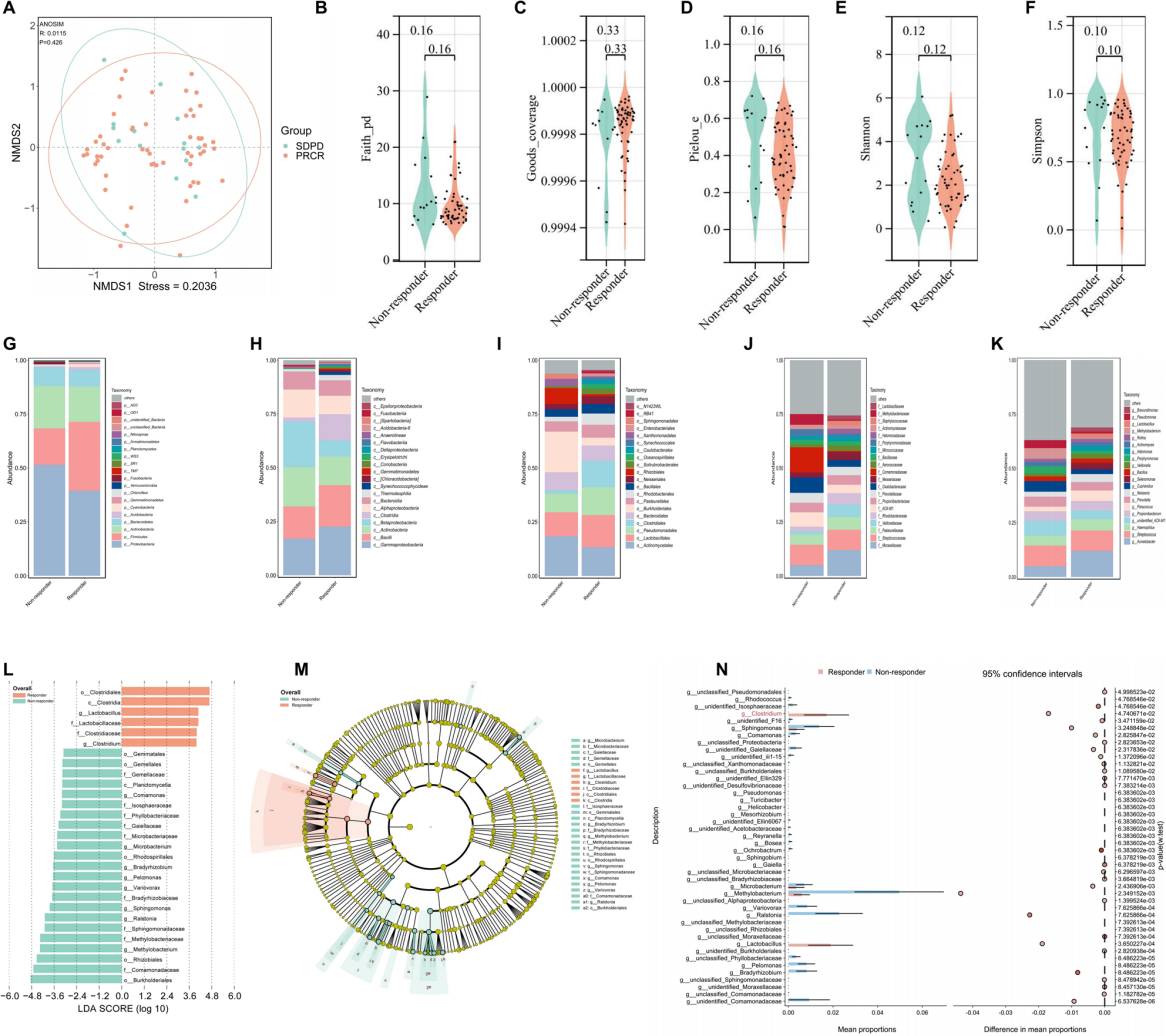
Comparison of tumor microbiota profiles between treatment responders and non-responders in SCLC. **A**. Intergroup distance display based on NMDS algorithm. **B-F**. Comparison of bacterial community α-diversity indices—including Faith_pd, Goods_coverage, Pielou_e, Shannon, and Simpson—between responders and non-responders. **G**. Bar plot comparing phylum-level tumor microbiota profiles between responders and non-responders. **H**. Bar plot comparing class-level tumor microbiota profiles between responders and non-responders. **I**. Bar plot comparing order-level tumor microbiota profiles between responders and non-responders. **J**. Bar plot comparing family-level tumor microbiota profiles between responders and non-responders. **K**. Bar plot comparing genus-level tumor microbiota profiles between responders and non-responders. **L**. Differential abundance of tumor microbiota between responders and non-responders, as determined by LEfSe analysis (LDA score > 2 and *P* < 0.05). **M**. Taxonomic Cladogram from LEfSe, depicting taxonomic association from between microbiome communities from groups of responders and non-responders. Each node represents a specific taxonomic type. Yellow nodes denote the taxonomic features that are not significantly differentiated across groups. Red nodes denote taxonomic types with higher abundance in the responder group and green nodes represent those more abundant in the non-responder group. N.Comparison of relative taxon abundance at the genus level between responders and non-responders, as determined by the Wilcoxon rank-sum test

Beta-diversity was evaluated using NMDS, indicating a high degree of similarity in the tumor microbiota composition between responders and non-responders (Fig. 5A). Besides, we compared bacterial community α-diversity indices—including Faith_pd, Goods_coverage, Pielou_e, Shannon, and Simpson—between responders and non-responders, and no significant differences were observed (Fig. 5B-F). The proportional abundance of the tumor microbiota was assessed at different taxonomic ranks, including phylum (Fig. 5G), class (Fig. 5H), order (Fig. 5I), family (Fig. 5J), and genus (Fig. 5K), for each group. Disparities in tumor microbiota abundance were observed across taxonomic ranks, with *Acinetobacter* and *Paracoccus* showing higher abundance in responders versus non-responders (Fig. 5K). To further pinpoint tumor microbiota with significant abundance differences between groups, LEfSe analysis was performed. The results indicated that *Clostridium* and *Lactobacillus* were significantly enriched in responders, whereas *Methylobacterium*, *Pelomonas*, *Ralstonia*, *Bradyrhizobium*, *Variovorax*, *Microbacterium*, *Comamonas*, and *Sphingomonas* were markedly enriched in non-responders (Fig. 5L–M). Consistent with these findings, the Wilcoxon rank-sum test yielded similar results (Fig. 5N).

**Fig. 6:**
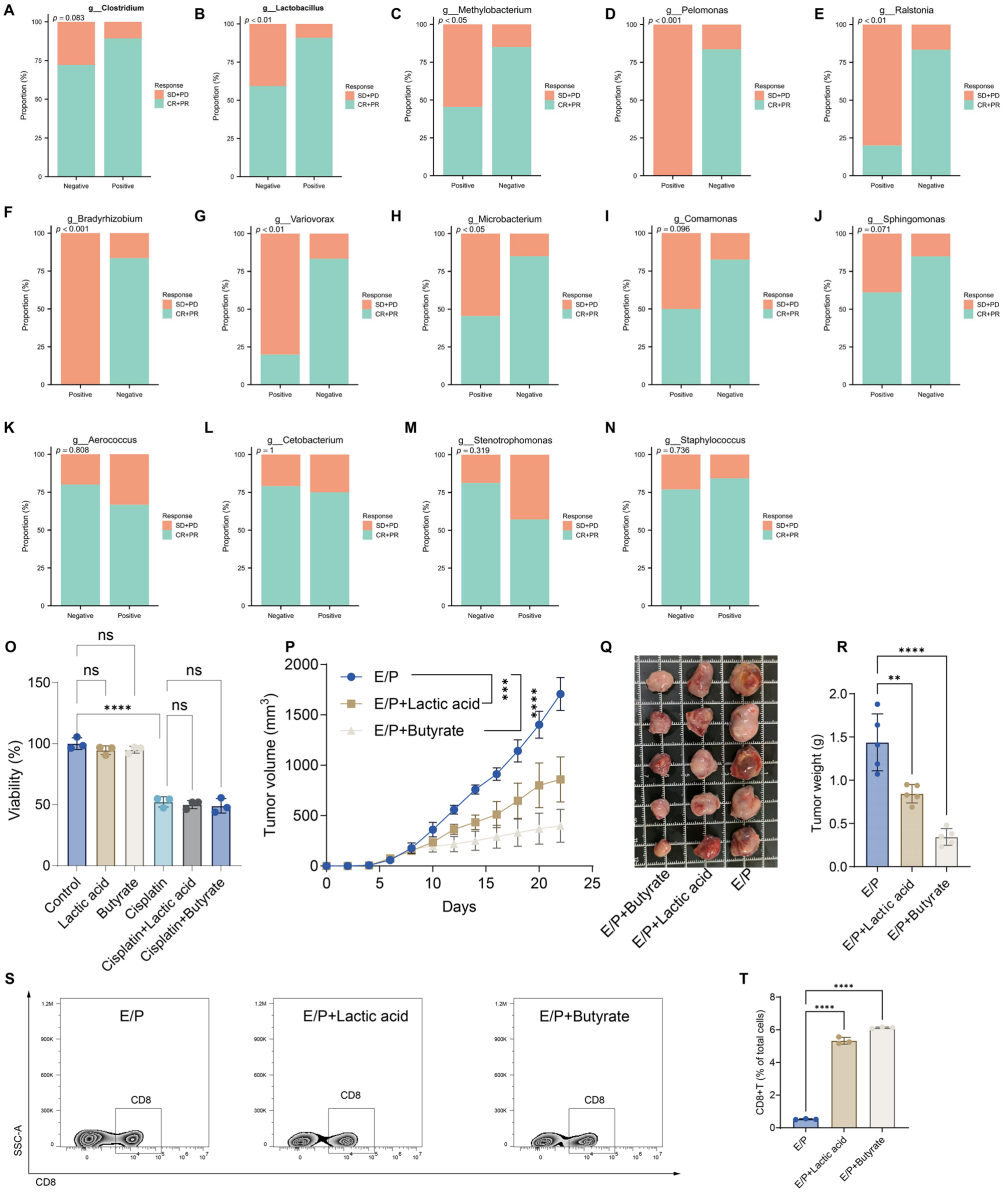
Comparison of treatment response by differential tumor microbiota status and subsequent functional validation of their metabolites in sensitizing SCLC to chemotherapy. **A**. Comparison of treatment response according to *Clostridium* status (negative vs. positive). **B**. Comparison of treatment response according to *Lactobacillus* status (negative vs. positive). **C**. Comparison of treatment response according to *Methylobacterium* status (negative vs. positive). **D**. Comparison of treatment response according to *Pelomonas* status (negative vs. positive). **E**. Comparison of treatment response according to *Ralstoni*a status (negative vs. positive). **F**. Comparison of treatment response according to *Bradyrhizobium* status (negative vs. positive). **G**. Comparison of treatment response according to *Variovorax* status (negative vs. positive). **H**. Comparison of treatment response according to *Microbacterium* status (negative vs. positive). **I**. Comparison of treatment response according to *Comamonas* status (negative vs. positive). **J**. Comparison of treatment response according to *Sphingomonas* status (negative vs. positive). **K**. Comparison of treatment response according to *Aerococcus* status (negative vs. positive). **L**. Comparison of treatment response according to *Cetobacterium* status (negative vs. positive). **M**. Comparison of treatment response according to *Stenotrophomonas* status (negative vs. positive). **N**. Comparison of treatment response according to *Staphylococcus* status (negative vs. positive). **O**. Cell viability of RPS1 cells was assessed by CCK-8 assay after 24 h of treatment with sodium butyrate (1 mM) or lactic acid (5 mM), both alone and in combination with cisplatin (2 µM). **P**. In vivo tumor growth curves. Immunocompetent mice bearing RPS1 tumors were treated with E/P (etoposide and cisplatin) chemotherapy, either alone or supplemented with sodium butyrate or sodium lactate. Tumor volumes were measured every two days (*n* = 5 per group). **Q**. Representative images of excised tumors. Gross morphology of tumors from each treatment group at the end of the experiment. **R**. Tumor weights at the experimental endpoint. Individual tumor weights were recorded upon sacrifice on Day 23. **S-T**. Flow cytometric profiling of tumor-infiltrating lymphocytes. Representative flow plots (S) and statistical quantification (T) showing the frequency of CD8 + T cells (gated on CD45 + CD3+ cells) within the tumor microenvironment

To further evaluate the impact of differential microbiota on treatment response, patients were stratified based on tumor microbiota status (negative vs. positive), and their treatment outcomes were compared. Regarding *Clostridium*, the response rate was higher in the positive group (89.3%) than in the negative group (72.1%); however, this difference did not reach statistical significance (*P* = 0.083; Fig. 6A). Analysis based on *Lactobacillus* status revealed a significantly higher response rate in the positive group (90.9%) compared to the negative group (59.3%) (*P* < 0.01; Fig. 6B). In contrast, a significantly lower response rate was observed in the *Methylobacterium*-positive group (45.5%) compared to the negative group (85.0%) (*P* < 0.05; Fig. 6C). Similar patterns were observed for *Pelomonas* (0% vs. 83.6%, *P* < 0.001; Fig. 6D), *Ralstonia* (20.0% vs. 83.3%, *P* < 0.01; Fig. 6E), *Bradyrhizobium* (0% vs. 83.6%, *P* < 0.01; Fig. 6F), *Variovorax* (20.0% vs. 83.3%, *P* < 0.01; Fig. 6G), and *Microbacterium* (45.5% vs. 85.0%, *P* < 0.05; Fig. 6H), where the response rate was significantly lower in the positive groups compared to their negative counterparts. For *Comamonas* (20.0% vs. 82.5%, *P* = 0.096; Fig. 6I) and *Sphingomonas* (61.1% vs. 84.9%, *P* = 0.071; Fig. 6J), a trend towards a reduced treatment response was observed in the positive groups compared to the negative groups. Additionally, we conducted statistical analysis on four tumor microbiota—*Aerococcus*, *Cetobacterium*, *Stenotrophomonas*, and *Staphylococcus*—which were previously found to significantly affect survival. In detail, *Aerococcus* (Fig. 6K), *Cetobacterium* (Fig. 6L), and *Stenotrophomonas* (Fig. 6M) positivity was associated with a weak—yet consistent—tendency for lower response rates. In contrast, *Staphylococcus*-positive status (Fig. 6N) correlated with a potential trend toward enhanced treatment response.

### Microbiota-derived metabolites potentiate chemotherapy efficacy

Since 16 S rRNA sequencing in this study did not identify the microbiota at the species level, we selected butyrate and lactic acid—the major metabolites of *Clostridium* and *Lactobacillus*—for functional exploration. As shown in Fig. 6O, butyrate and lactic acid did not exhibit obvious toxicity against SCLC, nor did they show a significant synergistic effect when combined with cisplatin. Furthermore, we conducted in vivo animal experiments, which revealed that compared to the chemotherapy-alone group, the addition of butyrate or lactic acid significantly inhibited tumor growth (Figs. 6P-Q). Consistently, tumor weight analysis demonstrated that the tumor weight in the combination groups was significantly lower than that in the chemotherapy-alone group (Fig. 6R). Finally, we performed flow cytometry to analyze the tumor immune microenvironment. The results indicated that the addition of either butyrate or lactic acid significantly increased the infiltration levels of CD8 + T cells (Figs. 6 S-T).

### Tumor microbiota profiles in smokers versus non-smokers

**Fig. 7:**
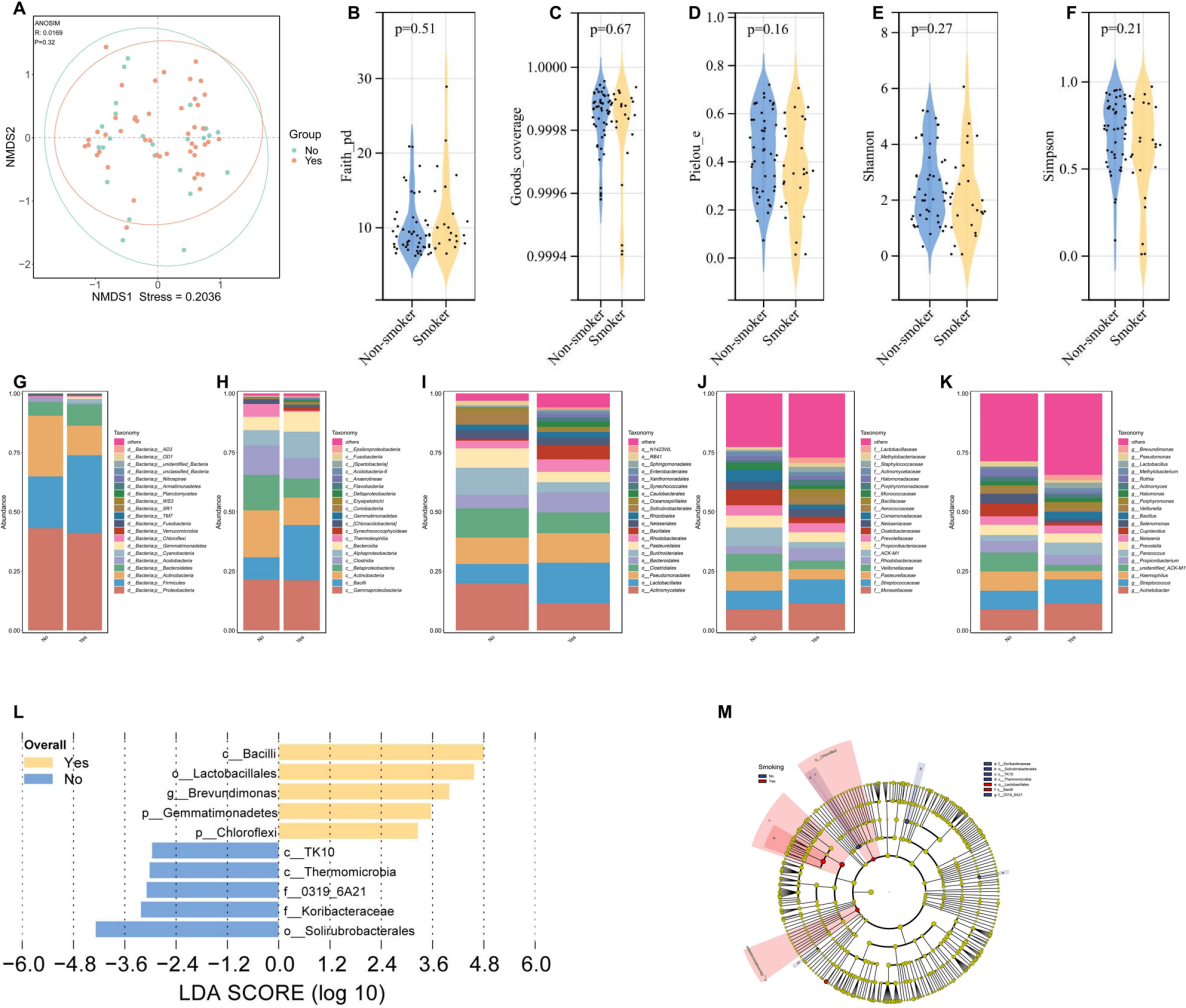
Tumor microbiota profiles in smokers versus non-smokers. **A**. Intergroup distance display based on NMDS algorithm. **B-F**. Comparison of bacterial community α-diversity indices—including Faith_pd, Goods_coverage, Pielou_e, Shannon, and Simpson—between smokers and non-smokers. **G-K**. Bar plot comparing tumor microbiota profiles across distinct ranks (phylum, class, order, family, and genus) between smokers and non-smokers. **L**. Differential abundance of tumor microbiota between smokers and non-smokers, as determined by LEfSe analysis (LDA score > 2 and *P* < 0.05). **M**. Taxonomic Cladogram from LEfSe, depicting taxonomic association from between microbiome communities from groups of smokers and non-smokers. Each node represents a specific taxonomic type. Yellow nodes denote the taxonomic features that are not significantly differentiated across groups. Red nodes denote taxonomic types with higher abundance in the smoker group and blue nodes represent those more abundant in the non-smoker group

Beta-diversity of tumor microbiota showed no clear separation between smokers and non-smokers (Fig. 7A). Similarly, no significant divergence in α-diversity was detected between the two groups (Figs. 7B-F). Comparative analyses of tumor microbiota abundance profiles were conducted between smokers and non-smokers across various taxonomic ranks. At the phylum level, the non-smoking group showed higher abundance of *Actinobacteria*, while the smoking group exhibited increased abundance of *Firmicutes* (Fig. 7G). Analyses at the class, order, family, and genus levels are provided in Fig. 7H, I and J, and 7K, respectively. To identify smoking-induced alterations in the tumor microbiota, LEfSe was performed. Significant enrichments were observed in smokers for *Lactobacillales*, *Brevundimonas*, and *Bacilli* compared to non-smokers. Conversely, the class *Thermomicrobia*, family *Koribacteraceae*, and order *Solirubrobacterales* were found to be significantly enriched in non-smokers (Fig. 7L-M). Collectively, these results suggest that smoking could impact clinical outcomes in cancer patients by altering the composition and function of the tumor microbiota.

### Construction of predictive models for treatment response and prognosis based on tumor microbiota

**Fig. 8:**
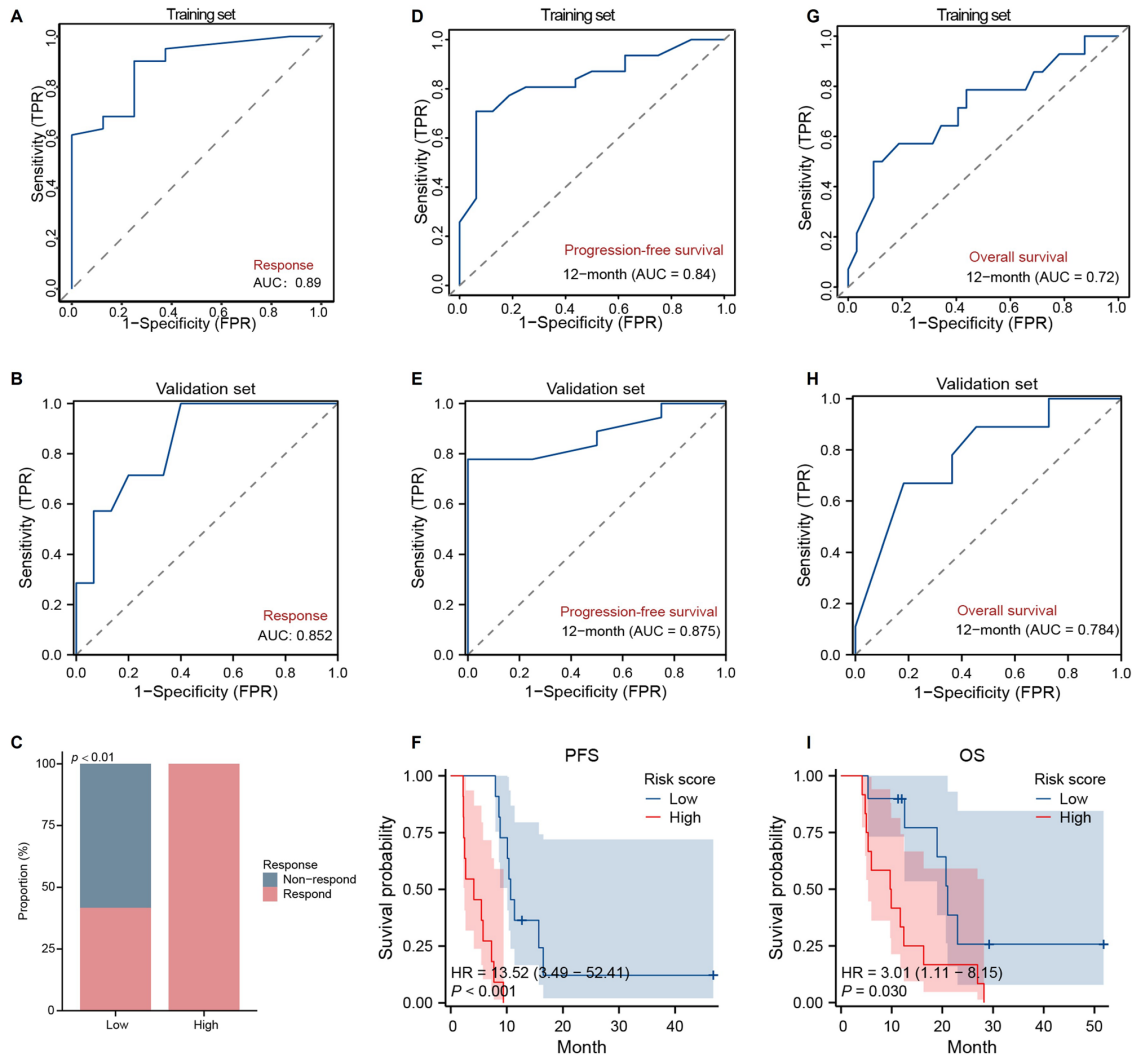
Construction of predictive models for treatment response and prognosis. **A**. Construction of treatment response prediction model based on multivariable logistic regression that integrated tumor microbiota (*Clostridium* and *Lactobacillus*) and clinical features (sex, age, smoking, stage, radiotherapy) using the training set (*n* = 49). **B**. Performance evaluation of the treatment response prediction model in the validation set (*n* = 22). **C**. Comparison of treatment response between high-risk and low-risk groups stratified by the treatment response prediction model–derived risk score in the validation set (*n* = 22). **D**. Progression-free survival (PFS) prediction model built by multivariable Cox regression integrating tumor microbiota (*Clostridium* and *Lactobacillus*) and clinical features (sex, age, smoking, stage, radiotherapy) in the training set (*n* = 49). **E**. Performance evaluation of the PFS prediction model in the validation set (*n* = 22). **F**. Comparison of PFS between high-risk and low-risk groups stratified by the PFS prediction model–derived risk score in the validation set (*n* = 22). **G**. Overall survival (OS) prediction model built by multivariable Cox regression integrating tumor microbiota (*Clostridium* and *Lactobacillus*) and clinical features (sex, age, smoking, stage, radiotherapy) in the training set (*n* = 49). **H**. Performance evaluation of the OS prediction model in the validation set (*n* = 22). **I**. Comparison of OS between high-risk and low-risk groups stratified by the OS prediction model–derived risk score in the validation set (*n* = 22)

Based on the comprehensive results from both survival and treatment response analyses, we incorporated *Clostridium* and *Lactobacillu*s—which significantly influenced both survival and therapeutic response—along with clinical features (sex, age, smoking, stage, radiotherapy) into the development of predictive models for treatment response and prognosis. Firstly, a treatment prediction response model was developed based on multivariable logistic regression in the training set. The model demonstrated excellent predictive performance with an area under the curve (AUC) of 0.89 (Fig. 8A). In the validation set, the model achieved an AUC of 0.852 (Fig. 8B), indicating good reliability and generalizability. Furthermore, we compared treatment responses between high- and low-risk groups—defined by the model-derived risk score—in the validation set. The response rate in the low-risk group was significantly higher than that in the high-risk group (100% vs. 41.7%, *P* < 0.01; Fig. 8C). Secondly, a 12-month PFS model was developed in the training set via multivariable Cox regression (AUC = 0.84; Fig. 8D) and subsequently validated in the validation set, showing strong predictive performance (AUC = 0.875; Fig. 8E). The model-derived risk score enabled significant stratification of patients into high- and low-risk groups. Those in the high-risk category exhibited a median PFS of 4.13 months, significantly shorter than the 10.67 months observed in the low-risk group (HR = 13.52, 95% CI: 3.49–52.41, *P* < 0.001; Fig. 8F). Thirdly, a 12-month OS prediction model was constructed using the training set based on multivariable Cox regression and validated on the validation set, yielding AUC values of 0.72 (Fig. 8G) and 0.78 (Fig. 8H), respectively, indicating good predictive performance. Similarly, the risk stratification based on this model revealed significant differences in survival. Patients in the high-risk category exhibited a median OS of 9.8 months, substantially shorter than the 21.033 months observed in the low-risk group (HR = 3.01, 95% CI: 1.11–8.15, *P* = 0.03; Fig. 8I).

## Discussion

With the advancement of sequencing technology, a growing body of evidence suggests the existence of tumor microbiota [15, 19, 20], which may represent another crucial component of the TME and play a significant role in regulating the TME, tumor initiation and progression, as well as treatment outcomes [21, 22]. Currently, the landscape of tumor microbiota in SCLC remains poorly characterized, and its potential impact on treatment response and clinical prognosis has not been systematically investigated. Here, we systematically characterized the tumor microbiota landscape in 71 SCLC tumor samples using 16 S rRNA sequencing and explored its relationship with patient prognosis and treatment response. Our findings revealed that distinct microbial communities were associated with differential clinical outcomes and therapeutic efficacy. These findings highlight the tumor microbiota as both a prognostic biomarker and suggest it may serve as a candidate therapeutic target, illuminating tumor–microbe crosstalk and paving the way for microbiota-based strategies to improve cancer therapy.

As an organ that interfaces directly with the external environment, the lung—and consequently lung cancer—may be more susceptible to colonization by diverse microbiota compared to malignancies originating in other organ sites. Indeed, multiple studies have confirmed the presence of tumor microbiota in lung cancer and its association with patient treatment response [23–25]. Ma et al. identified various microbial communities, including *Firmicutes*, *Proteobacteria*, and *Actinobacteria*, through 16 S rRNA sequencing of surgical specimens from stage I non-small cell lung cancer patients [25]. In our study, *Firmicutes*, *Proteobacteria*, and *Actinobacteria* were also identified. Based on 16 S rRNA sequencing results, taxonomic annotation was performed at multiple levels, revealing 28 phyla, 79 classes, 135 orders, 251 families, and 428 genera, which delineate a highly diverse microbial community in SCLC. Our study delivers the first comprehensive landscape of the tumor-resident microbiota in SCLC, establishing an essential resource for mechanistic dissection and translational exploitation.

*Clostridium* is a large and heterogeneous genus of Gram-positive, spore-forming, obligate anaerobic bacilli, with *Clostridium butyricum* as a predominant species, which primarily produces butyrate as its key metabolic product [26, 27]. Accumulating evidence from multiple pre-clinical and clinical studies has consistently demonstrated the tumor-suppressive potential of non-pathogenic *Clostridium* strains [28–32]. Xu et al. demonstrated that *Clostridium butyricum* facilitates MYC degradation via the ubiquitin–proteasome pathway, while concurrently downregulating the expression of thymidylate synthase and programmed cell death ligand 1, thereby overcoming 5-fluorouracil resistance and enhancing the efficacy of immunotherapy in colorectal cancer [30]. In the Phase I trial NCT01924689, a single intratumoral injection of *Clostridium novyi-NT* spores in refractory solid tumors yielded stable disease in 86% of patients, while systemic cytokine release and amplified tumor-specific T-cell responses provided proof-of-concept that anaerobic oncolysis is feasible and can potentiate antitumor immunity [32, 33]. Consistent with these findings, our analysis of 16 S rRNA sequencing data revealed a significant enrichment of *Clostridium* in SCLC. Patients harboring *Clostridium*-positive tumors demonstrated superior treatment responses and prolonged survival compared to their *Clostridium*-negative counterparts. To mechanistically validate these clinical observations, we evaluated the effects of butyrate, the primary metabolite of *Clostridium*. Interestingly, while butyrate exhibited no direct cytotoxicity against SCLC cells and failed to enhance cisplatin sensitivity in *vitro*, its systemic administration drastically potentiated the efficacy of E/P chemotherapy in vivo. Consistent with recent studies demonstrating that butyrate acts as a potent epigenetic orchestrator to enhance CD8 + T cell metabolic fitness and effector function [17, 34], our flow cytometry analysis revealed a massive intratumoral infiltration of CD8 + T cells following butyrate supplementation. These findings suggest that *Clostridium*-derived butyrate may prime the immune microenvironment to enhance chemo-sensitivity, potentially explaining why this bacterium is associated with favorable clinical outcomes in SCLC.

*Lactobacillus* is a genus of Gram-positive, non-spore-forming, facultatively anaerobic rod-shaped bacteria belonging to the phylum Firmicutes. Previous studies have demonstrated that gut microbe *Lactobacillus* plays a critical role in modulating tumor treatment response [35–37]. Lyu et al. demonstrated that *Lactobacillus johnsonii-FM1* restrains colorectal tumorigenesis by remodeling the microbiota and releasing vanillic acid, which suppresses Wnt/β-catenin signaling, induces cell-cycle arrest and apoptosis, and reduces tumor burden, highlighting a probiotic-based adjunct for colorectal cancer prevention and therapy [36]. More notably, a recent study revealed that tumor-resident *Lactobacillus iners*—an obligate L-lactate-producing bacterium—mediates chemoradiation resistance by rewiring tumor metabolism and activating lactate-driven signaling networks, thereby conferring poor prognosis across multiple cancer types [38]. In contrast, our data demonstrate that SCLC patients with *Lactobacillus*-positive tumors exhibited significantly improved therapeutic responses and prolonged survival compared to those with *Lactobacillus*-negative tumors. It should be noted that the 16 S rRNA sequencing approach employed in this study enabled taxonomic identification at the genus level but did not achieve sufficient resolution for species-level classification. This technical limitation may account for the discrepant findings between our study and previous reports, as different *Lactobacillus* species possess distinct metabolic capabilities and may exert opposing effects on tumor progression. To functionally dissect its role, we investigated lactic acid, the signature metabolite of *Lactobacillus*. Similar to butyrate, lactic acid exhibited no direct cytotoxicity against SCLC cells in vitro. However, systemic supplementation with lactic acid profoundly enhanced chemo-sensitivity in vivo, an effect accompanied by robust CD8 + T cell recruitment. This synergy aligns with an emerging paradigm shift in tumor immunology: under physiological pH, the lactate anion can serve as a primary carbon fuel for the tricarboxylic acid cycle and potently augment the stemness and anti-tumor efficacy of CD8 + T cells via histone lactylation [18]. Consequently, these in vivo findings provide compelling evidence that tumor-resident *Lactobacillus* may locally enrich the lactate pool to fuel anti-tumor immunity, rather than acting as a direct cytotoxic agent.

Previous studies have demonstrated that external environmental factors, particularly smoking, can modulate the composition and function of microbial communities—including the gut microbiota, respiratory tract microbiota, and intratumoral microbiota—thereby influencing tumor biological behavior and oncogenic properties [11, 23, 39, 40]. Greathouse et al. demonstrated that smoking selectively enriches *Acidovorax* (*Proteobacteria*) in TP53-mutant squamous-cell carcinomas thereby exemplifying microbiome–gene–exposure crosstalk that links cigarette smoke to lung cancer pathogenesis and unveiling candidate microbial biomarkers for early detection [41]. Our study showed no significant difference in microbial α-diversity between smokers and non-smokers; however, we identified specific taxa—such as *Brevundimonas* (genus) and *Gemmatimonadetes* (phylum)—that were significantly enriched in smokers. These findings suggest that, in addition to the previously established smoking–genome interaction mechanism influencing lung cancer, a smoking–tumor microbiota axis may represent a novel mechanism of lung carcinogenesis.

Collectively, with 16 S rRNA sequencing of 71 SCLC tumor samples, this study provides the first comprehensive characterization of the SCLC tumor microbiota landscape. Furthermore, two bacterial genera—*Clostridium* and *Lactobacillus*—were identified as being associated with higher response sensitivity to treatment and a favorable prognosis. These findings suggest their potential utility as predictive biomarkers for treatment response and as candidate targets for future microbiome-directed tumor interventions. Nevertheless, this study has limitations that warrant further investigation. First, 16 S rRNA sequencing restricts taxonomic resolution to the genus level. Given the species-level heterogeneity within *Clostridium* and *Lactobacillus*, therapeutic implications must be interpreted cautiously. To mitigate this, our functional assays focused on their conserved core metabolites (butyrate and lactic acid). Future targeted validation using species-specific qPCR or in situ hybridization is required to pinpoint the exact functional strains. Second, our reliance on 16 S rRNA sequencing restricts taxonomic resolution primarily to the genus level. Genera such as *Clostridium* and *Lactobacillus* comprise highly heterogeneous species that can possess divergent, and sometimes opposing, biological functions. To partially mitigate this limitation and provide functional resolution, our in vivo experiments focused on their universally recognized core metabolites (butyrate and lactic acid). Nevertheless, to move beyond correlative interpretations, future studies must employ deep shotgun metagenomics, combined with targeted validation using species-specific quantitative PCR or fluorescence in situ hybridization, to pinpoint the exact functional species and strains driving these clinical benefits in SCLC. Third, our cohort included biopsies from both primary tumors and metastatic sites (lymph nodes and liver). While the sample size limited our ability to conduct robust subgroup comparisons across all anatomical niches, future studies with larger cohorts are needed to determine if the tumor microbiota composition significantly diverges between primary and metastatic lesions in SCLC. Additionally, the low microbial biomass of tumors makes microbiome profiling susceptible to reagent contamination. Although our physical negative controls yielded insufficient DNA for sequencing, taxa enriched in non-responders (e.g., *Ralstonia*, *Bradyrhizobium*) are well-known potential contaminants. However, given the lung’s continuous environmental exposure, these taxa may also represent genuine inhaled colonizers. While their differential abundance implies a biological pattern, we cannot definitively rule out background noise. Future studies are required for further exploration.

## Conclusion

In conclusion, the tumor-resident microbiota constitutes a critical component of the SCLC tumor microenvironment, exerting profound influence on the therapeutic response and patient prognosis. In detail, *Clostridium* and *Lactobacillus*—two pivotal tumor-resident taxa significantly linked to enhanced therapeutic responses and favorable prognosis—indicate their potential as predictive biomarkers for treatment outcomes and patient prognosis, and highlight them as candidate targets for microbiome-directed therapeutic strategies against SCLC, which warrants further functional validation.

## Supplementary Information

Below is the link to the electronic supplementary material.

Supplementary Material 1

## Data Availability

All data used and/or analyzed in the study are available in the supplementary materials or from the corresponding author upon reasonable request.
