# Supplementary material for: Profiling the tumor-resident microbiota in small cell lung cancer and its influence on clinical outcomes
Source: J Transl Med. 2026 Apr 14;24:537. doi: 10.1186/s12967-026-08109-x (PMC13088536; doi:10.1186/s12967-026-08109-x)
Supplement: Supplementary file 1 — Supplementary Material 1 [file 12967_2026_8109_MOESM1_ESM.pdf]

Supplemental Material

Table S1.Multivariate Cox regression analyses to evaluate the independent prognostic value of *Aerococcus* for PFS in SCLC.

Variables	β	S.E	Z	*P*	HR (95%CI)
Sex					
Female					1.00 (Reference)
Male	-0.08	0.55	-0.15	0.879	0.92 (0.31 ~ 2.70)
Smoking					
No					1.00 (Reference)
Yes	0.73	0.34	2.12	**0.034**	2.07 (1.05 ~ 4.07)
Stage					
ES					1.00 (Reference)
LS	-0.55	0.54	-1.02	0.307	0.58 (0.20 ~ 1.66)
Radiotherapy					
No					1.00 (Reference)
Yes	-0.58	0.54	-1.07	0.283	0.56 (0.19 ~ 1.62)
Aerococcus					
Negative					1.00 (Reference)
Positive	0.73	0.48	1.51	0.13	2.08 (0.81 ~ 5.35)

Table S2.Multivariate Cox regression analysis to evaluate the independent prognostic value of *Cetobacterium* for PFS in SCLC.

Variables	β	S.E	Z	*P*	HR (95%CI)
Sex					
Female					1.00 (Reference)
Male	-0.23	0.54	-0.42	0.675	0.80 (0.28 ~ 2.29)
Smoking					
No					1.00 (Reference)
Yes	0.76	0.34	2.23	**0.026**	2.14 (1.10 ~ 4.19)
Stage					
ES					1.00 (Reference)
LS	-0.74	0.53	-1.39	0.164	0.48 (0.17 ~ 1.35)
Radiotherapy					
No					1.00 (Reference)
Yes	-0.44	0.55	-0.79	0.429	0.65 (0.22 ~ 1.91)
Cetobacterium					
Negative					1.00 (Reference)
Positive	0.64	0.55	1.16	0.245	1.90 (0.64 ~ 5.62)

Table S3.Multivariate Cox regression analysis to evaluate the independent prognostic value of *Clostridium* for PFS in SCLC.

Variables	β	S.E	Z	*P*	HR (95%CI)
Sex					
Female					1.00 (Reference)
Male	-0.39	0.54	-0.72	0.471	0.68 (0.23 ~ 1.95)
Smoking					
No					1.00 (Reference)
Yes	0.72	0.34	2.13	**0.033**	2.06 (1.06 ~ 4.01)
Stage					
ES					1.00 (Reference)
LS	-0.86	0.53	-1.63	0.103	0.42 (0.15 ~ 1.19)
Radiotherapy					
No					1.00 (Reference)
Yes	-0.41	0.53	-0.76	0.445	0.67 (0.23 ~ 1.89)
Clostridium					
Negative					1.00 (Reference)
Positive	-0.71	0.28	-2.55	**0.011**	0.49 (0.28 ~ 0.85)

Table S4.Multivariate Cox regression analysis to evaluate the independent prognostic value of *Lactobacillus* for PFS in SCLC.

Variables	β	S.E	Z	*P*	HR (95%CI)
Sex					
Female					1.00 (Reference)
Male	-0.42	0.56	-0.75	0.454	0.66 (0.22 ~ 1.97)
Smoking					
No					1.00 (Reference)
Yes	1.01	0.36	2.85	**0.004**	2.75 (1.37 ~ 5.52)
Stage					
ES					1.00 (Reference)
LS	-0.51	0.54	-0.94	0.346	0.60 (0.21 ~ 1.74)
Radiotherapy					
No					1.00 (Reference)
Yes	-0.61	0.55	-1.1	0.27	0.55 (0.19 ~ 1.60)
Lactobacillus					
Negative					1.00 (Reference)
Positive	-0.76	0.28	-2.67	**0.008**	0.47 (0.27 ~ 0.82)

Table S5.Multivariate Cox regression analysis to evaluate the independent prognostic value of *Staphylococcus* for PFS in SCLC.

Variables	β	S.E	Z	*P*	HR (95%CI)
Sex					
Female					1.00 (Reference)
Male	0.11	0.55	0.2	0.842	1.11 (0.38 ~ 3.25)
Smoking					
No					1.00 (Reference)
Yes	0.79	0.33	2.37	**0.018**	2.20 (1.15 ~ 4.22)
Stage					
ES					1.00 (Reference)
LS	0.03	0.57	0.06	0.955	1.03 (0.34 ~ 3.18)
Radiotherapy					
No					1.00 (Reference)
Yes	-1.53	0.62	-2.47	**0.014**	0.22 (0.06 ~ 0.73)
Staphylococcus					
Negative					1.00 (Reference)
Positive	-1.22	0.35	-3.52	**<.001**	0.29 (0.15 ~ 0.58)

Table S6.Multivariate Cox regression analysis to evaluate the independent prognostic value of *Stenotrophomonas* for PFS in SCLC.

Variables	β	S.E	Z	*P*	HR (95%CI)
Sex					
Female					1.00 (Reference)
Male	-0.47	0.53	-0.88	0.377	0.62 (0.22 ~ 1.77)
Smoking					
No					1.00 (Reference)
Yes	0.78	0.34	2.29	**0.022**	2.19 (1.12 ~ 4.28)
Stage					
ES					1.00 (Reference)
LS	-1.35	0.61	-2.22	**0.026**	0.26 (0.08 ~ 0.85)
Radiotherapy					
No					1.00 (Reference)
Yes	0.13	0.61	0.22	0.827	1.14 (0.34 ~ 3.79)
Stenotrophomonas					
Negative					1.00 (Reference)
Positive	1.2	0.51	2.37	**0.018**	3.33 (1.23 ~ 9.03)

Table S7.Multivariate Cox regression analysis to evaluate the independent prognostic value of *Aerococcus* for OS in SCLC.

Variables	β	S.E	Z	*P*	HR (95%CI)
Sex					
Female					1.00 (Reference)
Male	-0.29	0.62	-0.47	0.638	0.75 (0.22 ~ 2.53)
Smoking					
No					1.00 (Reference)
Yes	0.97	0.4	2.44	**0.014**	2.65 (1.21 ~ 5.78)
Stage					
ES					1.00 (Reference)
LS	-0.27	0.61	-0.44	0.657	0.76 (0.23 ~ 2.51)
Radiotherapy					
No					1.00 (Reference)
Yes	-0.66	0.61	-1.09	0.277	0.52 (0.16 ~ 1.70)
Aerococcus					
Negative					1.00 (Reference)
Positive	0.26	0.47	0.54	0.588	1.29 (0.51 ~ 3.28)

Table S8.Multivariate Cox regression analysis to evaluate the independent prognostic value of *Cetobacterium* for OS in SCLC.

Variables	β	S.E	Z	*P*	HR (95%CI)
Sex					
Female					1.00 (Reference)
Male	-0.36	0.61	-0.59	0.552	0.70 (0.21 ~ 2.30)
Smoking					
No					1.00 (Reference)
Yes	1.05	0.4	2.62	**0.009**	2.85 (1.30 ~ 6.24)
Stage					
ES					1.00 (Reference)
LS	-0.33	0.6	-0.55	0.579	0.72 (0.22 ~ 2.31)
Radiotherapy					
No					1.00 (Reference)
Yes	-0.68	0.62	-1.09	0.274	0.51 (0.15 ~ 1.71)
Cetobacterium					
Negative					1.00 (Reference)
Positive	-0.15	0.56	-0.27	0.788	0.86 (0.29 ~ 2.57)

Table S9.Multivariate Cox regression analysis to evaluate the independent prognostic value of *Clostridium* for OS in SCLC.

Variables	β	S.E	Z	*P*	HR (95%CI)
Sex					
Female					1.00 (Reference)
Male	-0.37	0.61	-0.61	0.544	0.69 (0.21 ~ 2.29)
Smoking					
No					1.00 (Reference)
Yes	1.01	0.39	2.56	**0.01**	2.75 (1.27 ~ 5.95)
Stage					
ES					1.00 (Reference)
LS	-0.34	0.59	-0.57	0.569	0.71 (0.22 ~ 2.28)
Radiotherapy					
No					1.00 (Reference)
Yes	-0.64	0.62	-1.03	0.304	0.53 (0.16 ~ 1.78)
Clostridium					
Negative					1.00 (Reference)
Positive	-0.04	0.3	-0.14	0.889	0.96 (0.53 ~ 1.73)

Table S10.Multivariate Cox regression analysis to evaluate the independent prognostic value of *Lactobacillusm* for OS in SCLC.

Variables	β	S.E	Z	*P*	HR (95%CI)
Sex					
Female					1.00 (Reference)
Male	-0.41	0.61	-0.66	0.509	0.67 (0.20 ~ 2.22)
Smoking					
No					1.00 (Reference)
Yes	1.06	0.39	2.71	**0.007**	2.89 (1.34 ~ 6.22)
Stage					
ES					1.00 (Reference)
LS	-0.25	0.61	-0.41	0.678	0.78 (0.24 ~ 2.55)
Radiotherapy					
No					1.00 (Reference)
Yes	-0.64	0.61	-1.04	0.297	0.53 (0.16 ~ 1.76)
Lactobacillus					
Negative					1.00 (Reference)
Positive	-0.25	0.31	-0.79	0.432	0.78 (0.42 ~ 1.45)

Table S11.Multivariate Cox regression analysis to evaluate the independent prognostic value of *Staphylococcus* for OS in SCLC.

Variables	β	S.E	Z	*P*	HR (95%CI)
Sex					
Female					1.00 (Reference)
Male	-0.22	0.61	-0.36	0.721	0.80 (0.24 ~ 2.68)
Smoking					
No					1.00 (Reference)
Yes	0.97	0.38	2.53	**0.011**	2.63 (1.24 ~ 5.58)
Stage					
ES					1.00 (Reference)
LS	0.04	0.65	0.05	0.957	1.04 (0.29 ~ 3.69)
Radiotherapy					
No					1.00 (Reference)
Yes	-1.03	0.67	-1.53	0.125	0.36 (0.10 ~ 1.33)
Staphylococcus					
Negative					1.00 (Reference)
Positive	-0.51	0.36	-1.39	0.163	0.60 (0.29 ~ 1.23)

Table S12.Multivariate Cox regression analysis to evaluate the independent prognostic value of *Stenotrophomonas* for OS in SCLC.

Variables	β	S.E	Z	*P*	HR (95%CI)
Sex					
Female					1.00 (Reference)
Male	-0.41	0.6	-0.69	0.491	0.66 (0.20 ~ 2.14)
Smoking					
No					1.00 (Reference)
Yes	0.94	0.4	2.36	**0.018**	2.56 (1.17 ~ 5.58)
Stage					
ES					1.00 (Reference)
LS	-0.48	0.6	-0.79	0.429	0.62 (0.19 ~ 2.02)
Radiotherapy					
No					1.00 (Reference)
Yes	-0.44	0.65	-0.67	0.501	0.65 (0.18 ~ 2.30)
Stenotrophomonas					
Negative					1.00 (Reference)
Positive	0.42	0.47	0.9	0.37	1.52 (0.61 ~ 3.78)

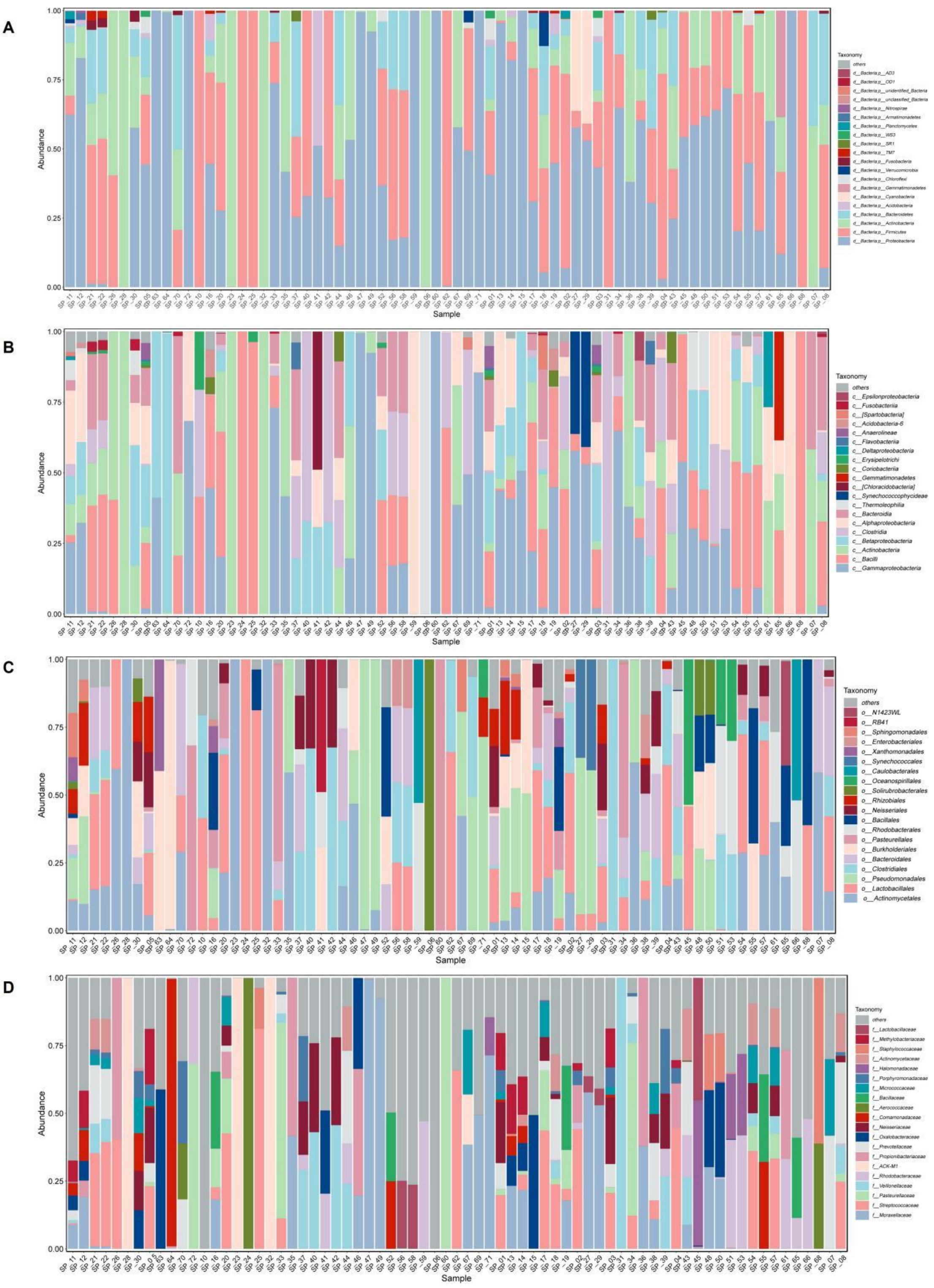

Figure S1.Compositional profiling of the top 20 taxa in the tumor microbiota at multiple taxonomic levels.

A.Tumor microbiota composition profiling of the top 20 taxa at phylum level across all samples.

B.Tumor microbiota composition profiling of the top 20 taxa at class level across all samples.

C.Tumor microbiota composition profiling of the top 20 taxa at order level across all samples.

D.Tumor microbiota composition profiling of the top 20 taxa at family level across all

samples.

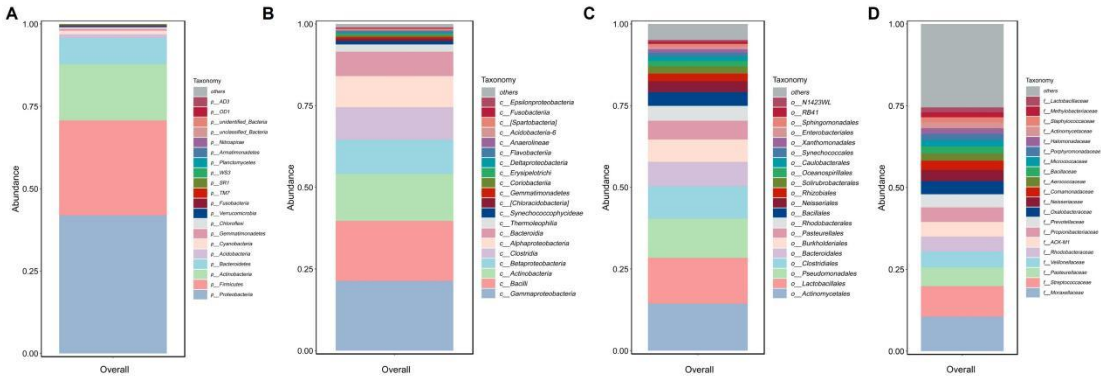

Figure S2.Profiling the most abundant taxa in the SCLC tumor microbiota at multiple taxonomic levels.

A.Relative abundance of the top 20 phylum-level taxa in the tumor microbiota from 71 enrolled SCLC patients (pooled data).

B.Relative abundance of the top 20 class-level taxa in the tumor microbiota from 71 enrolled SCLC patients (pooled data).

C.Relative abundance of the top 20 order-level taxa in the tumor microbiota from 71 enrolled SCLC patients (pooled data).

D.Relative abundance of the top 20 family-level taxa in the tumor microbiota from 71 enrolled SCLC patients (pooled data).

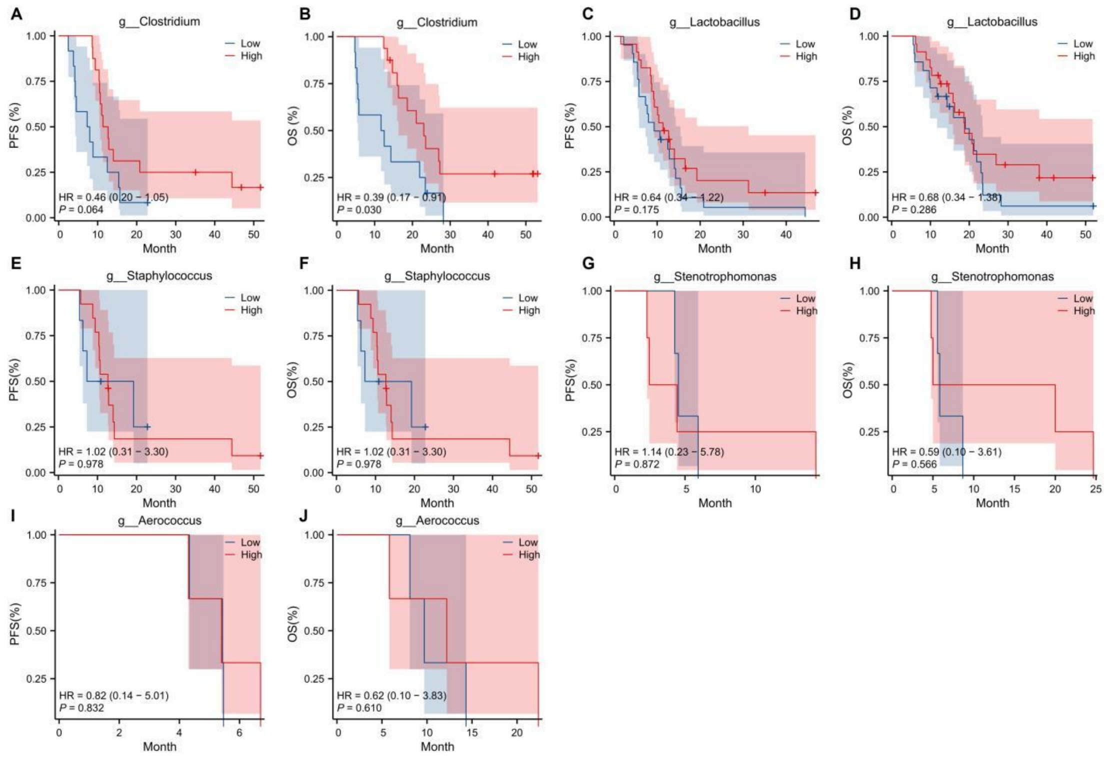

Figure S3.Survival analysis based on the abundance of differential tumor microbiota (high vs. low).

A.Comparison of progression-free survival (PFS) in Clostridium-positive SCLC patients stratified by abundance level (high vs. low).

B.Comparison of overall survival (OS) in Clostridium-positive SCLC patients stratified by abundance level (high vs. low).

C.Comparison of PFS in Lactobacillus-positive SCLC patients stratified by abundance level (high vs. low).

D.Comparison of OS in Lactobacillus-positive SCLC patients stratified by abundance level (high vs. low).

E.Comparison of PFS in Staphylococcus-positive SCLC patients stratified by abundance level (high vs. low).

F.Comparison of OS in Staphylococcus-positive SCLC patients stratified by abundance level (high vs. low).

G.Comparison of PFS in Stenotrophomonas-positive SCLC patients stratified by abundance level (high vs. low).

H.Comparison of OS in Stenotrophomonas-positive SCLC patients stratified by abundance level (high vs. low).

I.Comparison of PFS in Aerococcus-positive SCLC patients stratified by abundance level (high vs. low).

J.Comparison of OS in Aerococcus-positive SCLC patients stratified by abundance level (high vs. low).

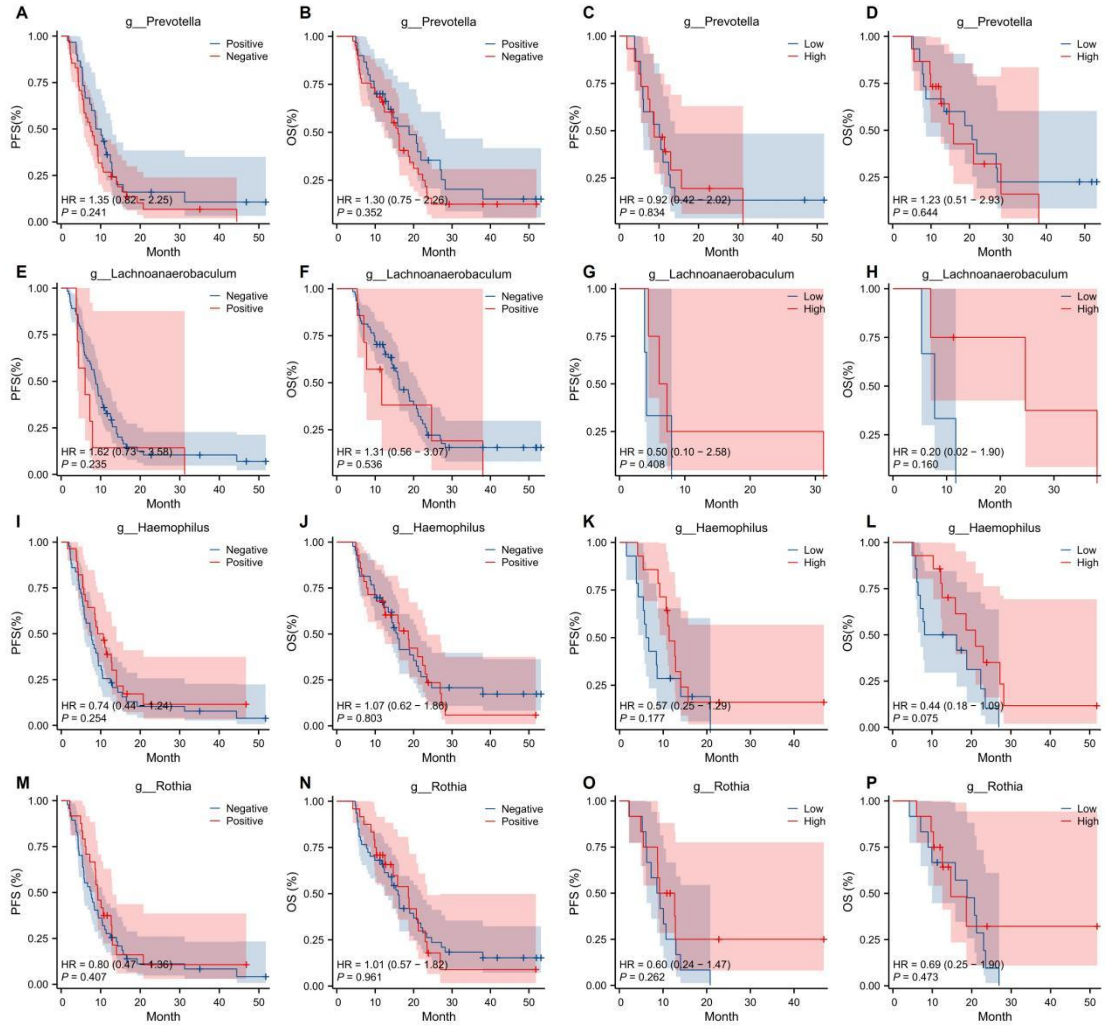

Figure S4.Survival analysis based on the other differential tumor microbiota.

A-B.PFS and OS comparison according to Prevotella status (negative vs. positive).

C-D.PFS and OS comparison in Prevotella-positive SCLC patients stratified by abundance level (high vs. low).

E-F.PFS and OS comparison according to Lachnoanaerobaculum status (negative vs. positive).

G-H.PFS and OS comparison in Lachnoanaerobaculum-positive SCLC patients stratified by abundance level (high vs. low).

I-J.PFS and OS comparison according to Haemophilus status (negative vs. positive).

K-L.PFS and OS comparison in Haemophilus-positive SCLC patients stratified by abundance level (high vs. low).

M-N.PFS and OS comparison according to Rothia status (negative vs. positive).

O-P.PFS and OS comparison in Rothia-positive SCLC patients stratified by abundance level (high vs. low).
